# Scientific Appraisal and Therapeutic Properties of Plants Utilized for Veterinary Care in Poonch District of Jammu and Kashmir, India

**DOI:** 10.3390/biology11101415

**Published:** 2022-09-28

**Authors:** Zishan Ahmad Wani, Adil Farooq, Sobia Sarwar, Vikram S. Negi, Ali Asghar Shah, Bikarma Singh, Sazada Siddiqui, Shreekar Pant, Huda Alghamdi, Mahmoud Mustafa

**Affiliations:** 1 Conservation Ecology Lab, Department of Botany, Baba Ghulam Shah Badshah University, Rajouri 185234, Jammu and Kashmir, India; 2 Department of Botany, Lahore College for Women University, Lahore 05422, Pakistan; 3 Center for Biodiversity Conservation and Management, G. B. Pant, National Institute of Himalayan Environment, Almora 263145, Uttarakhand, India; 4 School of Biosciences and Biotechnology, Baba Ghulam Shah Badshah University, Rajouri 185234, Jammu and Kashmir, India; 5 Botanical Garden Division, CSIR National Botanical Research Institute (NBRI), Lucknow 226001, Uttar Pradesh, India; 6 Department of Biology, College of Science, King Khalid University, Abha 61441, Saudi Arabia; 7 Centre for Biodiversity Studies, Baba Ghulam Shah Badshah University, Rajouri 185234, Jammu and Kashmir, India

**Keywords:** animal care, informant consensus, Jaccard index, reverse pharmacology, phytochemicals

## Abstract

**Simple Summary:**

In the rural areas of the Himalaya, ethnobotanical knowledge is crucial for preserving plant diversity and treating a variety of diseases. Ethno-veterinary medicines can provide leads for drug development, but probably a more practical and lucrative exercise would be to develop a preferred preparation by pharmacological research, and the ensuing medication can be returned to the society with extra impact. Further, this valuable knowledge base has now become obsolete due to industrialization, urbanization and, above all, lack of recognition by the younger generations. Therefore, there is a dire need to review, document and authenticate the valuable traditional knowledge of ethno-medicinal plants for human welfare. By comprehending the traditional knowledge system, the current study conducted in Jammu and Kashmir, India, could serve as a pilot to document the sustainable exploitation of regularly utilized bioresources and would provide crucial leads for the manufacturing of pharmaceuticals and medicines.

**Abstract:**

The importance of traditional and indigenous knowledge is acknowledged on a worldwide scale for its coexistence principles and sustainable use techniques. In view of this, the present study is an attempt to document the ethno-veterinary plants used by the tribal communities of Western Himalaya. This study also provides the scientific validation of herbal medicines used in ethno-veterinary practices through a reverse pharmacological approach. A total of 59 informants were selected through a non-probability sampling method. Detailed information on the medicinal plants used in ethno-veterinary practices along with their habits and habitats, part/s used, remedy preparation methods, additives/ingredients used during preparation and administration, dosages administered, and route of administration was collected. Data was analyzed for the Relative Frequency of Citations (RFC), Use Values (UV), Informant Consensus Factor (ICF), and Jaccard Index (JI). Further, a reverse pharmacological approach was used for scientific validations of the documented herbal knowledge of plant species. During the study, 56 plant species belonging to 54 genera and 39 families were documented. Asteraceae was the dominant family followed by Lamiaceae, Amaranthaceae and Fabaceae. Life forms were dominated by herbaceous species and leaves were the most common plant parts used. The highest Relative Frequency of Citations (RFC) and Use Values (UV) were recorded for *Brassica rapa* L. (Brassicaceae). The Pearson correlation coefficient between RFC and UV shows a strong positive correlation between the proportion of uses of a plant species within a sample of informants and the number of times that a particular use of a plant species was mentioned by the informant. Studies of the biological activity of ethno-veterinary plants can provide clues of promising leads for the isolation and identification of useful compounds that may be developed into pharmaceuticals for human welfare.

## 1. Introduction

India has one of the primeval and sophisticated healthcare systems with a history of more than 5000 years [1]. This priceless information came together over the course of many centuries based on different traditional healthcare systems such as Rigveda, Atharveda, and several post Vedic treatises such as Charakasamhita, Dhanwanthari, and Nighantu [2]. Materia Medica provides a huge knowledge base of traditional healthcare systems of India [3]. Approximately, 25,000 plant-based formulations are used as traditional healthcare systems by different rural communities of India [4], and out of these, only 5–10% have been validated scientifically [3]. In various communities across India, the use of plant-based formulations has long been a crucial component in the treatment of various illnesses and has led manufacturers to produce a large number of medication candidates that are currently widely used in commercial markets [5]. However, various ethnomedicinal plants used in Indian healthcare systems have been investigated scientifically for nearly three decades [6]. Since then, the Government of India has made several attempts to investigate the possibility of evaluating these systems for their therapeutic potential as they were originally practiced, as well as to generate data to include them in national healthcare programs. This is due to the growing interest worldwide in adopting and studying traditional systems and in utilizing their potential from various healthcare perspectives [7]. According to the WHO report (2018), 66% of India’s population resides in rural areas with a large dependency on agriculture. In the Himalayan region, the rearing of animals to supplement the family income and sustain crop production constitutes an important component of the rural economy of the region [8,9,10]. In the mixed crop–livestock farming systems, livestock and food production systems are closely integrated in the Himalaya [11]. The Himalayan region’s rural economy is based mostly on animal husbandry, and the growth of this industry could raise the standard of living in rural areas. Farmers look after their livestock by using ethno-veterinary practices [12]. To keep domestic animals healthy and productive, ethno-veterinary remedies are widely and very successfully utilized for basic healthcare. People in remote rural areas continue to rely heavily on herbal and common domestic remedies to treat veterinary ailments. The system comprises conventional beliefs, knowledge, expertise, approaches, practices and traditions of a particular society [13,14]. Through a process of experience spanning hundreds of years, the traditional communities have identified the folk knowledge of ethno-veterinary medicine and its relevance. One of the most crucial prevailing systems in the region where modern veterinary healthcare facilities are scarce or in extremely bad condition is the traditional method of treatment [15]. Traditional herbalists (Pashu Vaidyas) pass down their native knowledge of the veterinary healthcare system orally from one generation to the next [16,17]. Thus, documentation of ethno-veterinary medicinal knowledge can generate leads for drug development, but on the basis of modern scientific approaches, there is inadequate data available on the evaluation and validation of this folk wisdom. One of the modern scientific approaches to validate the traditional knowledge of ethnic groups is reverse pharmacology. It is a multidisciplinary procedure for connecting traditional knowledge bases to promising research strategies, tools, technologies and innovations [18]. A notable component of this methodology is the blending of information gained from traditional or folk medicine with the advanced technology to guarantee better and more secure leads [19]. The attempt to study firm findings would assist not only in the recognition of the candidate drugs but also in the understanding of their underlying molecular mechanisms [20]. Reverse pharmacology has an immense scope to evaluate the efficacy and quality of traditionally used medicines clinically and, furthermore, to identify new drugs from the natural products used in traditional medicinal systems since times immemorial [18].

In the Himalayan region, people have strong a belief and faith in traditional herbal medicinal practices for healthcare, and therefore, the plants have immense cultural and medicinal significance within the region [21]. Nomadic tribes and pastoral communities residing within the Northwest and Trans-Himalaya are reputed to have mastered their conventional practices and knowledge regarding the ethno-medicinal and ethno-veterinary utilization of plants [22,23]. Jammu and Kashmir (J&K) is home to several tribal communities that are scattered throughout the region and form an inherent part of its culture and tradition [24]. The Gujjars and Bakerwals, seminomadic and nomadic tribes respectively, are the third-biggest ethnic groups in J&K, constituting more than 11.9% of the total population, and have maintained their culture and heritage throughout the ages [25]. These tribals depend on cattle rearing for their livelihood, and since they feed their livestock in the upper reaches of mountain terrain, they do not have easy access to modern veterinary medicine or doctors. They depend solely on folk medicine and herbal remedies for treating animal diseases. Oral transmission has been the only medium through which the traditional knowledge regarding the use of plants for healing purposes has been passed from generation to generation [26]. Lack of recognition by the younger generation has led to the decline of this valuable knowledge-base, and now it has become obsolete [27]. Furthermore, the indigenous knowledge of the use of lesser-known plants is also rapidly declining. Many researchers have documented the ethno-medicinal plants of J&K [28,29,30,31,32,33,34,35,36]; however, very limited attempts have been made to document the traditional herbal knowledge of veterinary systems. There is a dire need to review and document the valuable traditional knowledge regarding the use of ethno-veterinary plants, especially in remote and far-flung areas. In view of this, the present study aimed to provide a contemporary compilation of medicinal plants used for the treatment of animal diseases by the tribal communities of the district Poonch, Jammu and Kashmir, through quantitative ethnobotanical analysis. Further, the study attempted to validate the remedies through reverse pharmacological correlations.

## 2. Materials and Methods

### 2.1. Study Area

**Figure 1:**
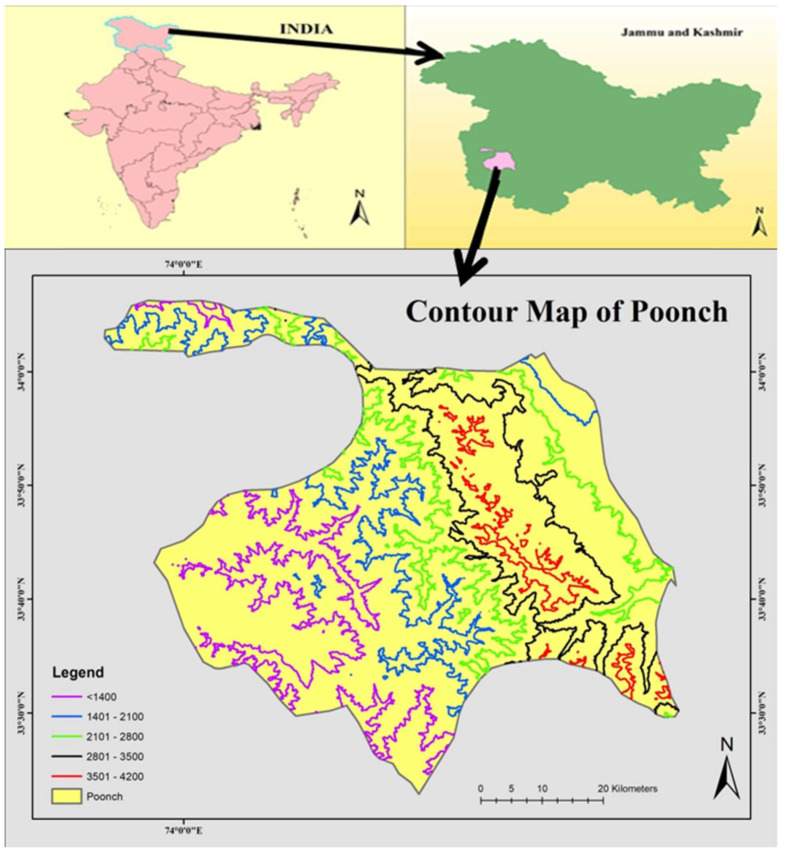
Map showing the location of the study area.

Poonch, renowned as ‘Mini Kashmir’ is one of the far-flung and backward districts of J&K situated on the Line of Control (LOC) located at 33.77° N and 74.1° E (Figure 1), and is delimited by the Actual Line of Control (ALC) from three sides. The Pir Panjal range of mountains separates Poonch valley from the Kashmir valley by the Pir Panjal Range. It is bordered by Baramulla, Budgam, Shopian and Kulgam Districts of Kashmir in the north east, Rajouri in the south and Azad Kashmir (Pakistan) in the west. Topographically, Poonch district is hilly and mountainous, exclusive of a few low-lying valleys. The vegetation of the study area is largely influenced by monsoon rainfall and varies from the humid zone to the temperate zone. Although people from many different linguistic groups live in the area, Gujjars and Bakerwals predominate. Both tribes have a similar ethnic make-up, speak the same language (Gojri), and rely on the same ecology to meet their daily requirements. The only distinction is that Gujjars raise buffalo and cows, whilst Bakerwals raise sheep and goats. The economic condition of people residing in the study area is not satisfactory and depends on herbal medicines for human and animal health care. Both groups have access to herbal healers to treat their common health issues, and oral transmission is the sole way this traditional knowledge is passed along.

### 2.2. Data Collection

A field assessment, which involved plant collection, photography, and data recording, was carried out between March 2019 and December 2020 following Heinrich et al. [37]. A total of 59 informants were selected through non-probability sampling, using convenience sampling methods based on easy access, availability, and relevance of informants. Out of the total 59 informants, 38 were males and 21 were females. The age group of the informants varies from 20 to 70 years, with a different level of education, from illiterate to above higher secondary level. The information of the respondents has been collected through questionnaires and direct interviews. Prior to interviewing and having discussions, formal written ethical consent for the study was obtained from the local tribal committee (vide number SPHC-B/108; Dated 19 February 2019) and oral consent from all participating informants was also given. Open semi-structured questionnaires developed by following Edwards et al. [38] with some modifications were used for collecting ethnobotanical data, as this approach permits several respondents to be cross-examined in a comparatively short period by posing the same questions within a flexible structure. Interviews were based on a checklist of questions set earlier in the English language and concurrently translated into Gojri, the local language, following Negi et al. [39]. Interviews focused on the demographic features of participants, including gender, age, matrimonial status, educational background, and the extent of time that an informant spends in the study area in a year. The foremost segment of the interviews focused on the local names of medicinal plants used, their life forms and habitats, part/s used, modes of remedial preparations, additives and ingredients used in the preparations, and dosages required. Besides the collection of data in written form, photographs and audio recordings were also taken for thorough analysis of the data and later verification following Ribeiro et al. [40].

Local guides/personnel were hired for the collection of plant specimens from the field. Fresh plant samples were collected from the field and were pressed, dried, sprayed with 1% mercuric chloride (preservative) and mounted on herbarium sheets following routine herbarium practices [41]. Collected plant samples were identified following regional floras [42,43,44], and the botanical nomenclature of the collected species was authenticated using the Plants of the World Online database (http://www.plantsoftheworldonline.org accessed on 21 March 2022).

### 2.3. Quantitative Data Analysis

The Microsoft Office Excel spreadsheet (2010, Microsoft Corporation, Redmond, WA, USA) was used for data scrutinization, computation, and drawing graphs. Some of the graphs were prepared using ‘*ggplots2*’ package in R statistical software (v 4.0.3; R Development Core Team 2021). The following quantitative and similarity metrics were used to analyze the data:

(A). *Relative Frequency of Citation* (RFC): RFC was calculated by using the following formula

**Figure 2:**
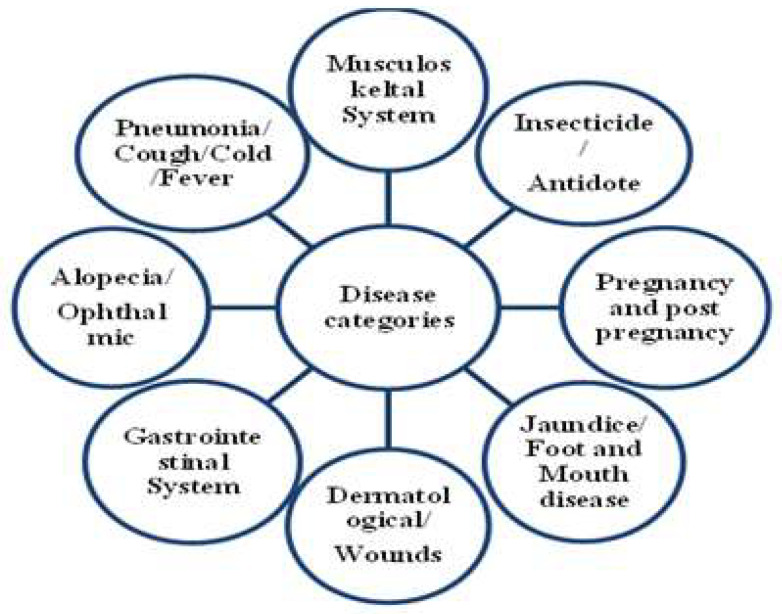
Disease categories for calculation of ICF.

RFC=FC/N

where FC is the number of informants reporting the use of a particular species and N is the total number of informants.

(B). *Use Value* (UV): Use value is an index in ethnobotany that has been commonly used to enumerate the relative significance of useful plants [45,46]. It is the relative importance of a particular plant species used by the indigenous inhabitants and was calculated following Phillips et al. [47]

$$\mathrm{UV}=\frac{\sum \mathrm{Ui}}{N}$$

where ‘Ui’ is the number of use-reports mentioned by each participant for the particular species and ‘N’ is the total number of informants. The use value ranges from 0 to 1. UV does not help in distinguishing whether a plant is used for single or multiple purposes [48].

(C). *Informant Consensus Factor* (ICF): To assess consistency of familiarity with the medicinal plants, the Informant Consensus Factor (ICF) was used by following [49]. Prior to analysis, all the ailments were categorized following Heinrich et al. [49] and Bhatia et al. [28]. The ICF was calculated by using the formula

$$\mathrm{ICF}=\frac{n_{\mathrm{ur}}-n_{t}}{n_{\mathrm{ur}}-1}$$

where ‘n_ur_’ is the number of use reports and ‘n_t_’ is the number of taxa utilized for a particular use category by all informants. ICF values range from 0.00 to 1.00. When only one or a few taxa are accounted to be used by a high proportion of informants for curing a particular illness, ICF values are higher, whereas low ICF values indicate that informants disagree about which plant to use and prefer different plants for curing a particular disease [49]. In the present study, eight disease categories were identified (Figure 2) and the ICF was calculated for each disease category.

(D). *Jaccard Index* (JI): In order to compare the present study with similar previously published literature and to access comparison of knowledge among different communities, the Jaccard Index was calculated using the following formula [50,51,52]

$$\mathrm{JI}=\frac{c\times 100}{\left(a+b\right)-c}$$

where “a” is the number of species of area A (our study area); “b” is the number of species of the neighboring area B; “c” is the number of species common to both A and B.

### 2.4. Statistical Analysis

The Pearson correlation between the RFC and UV was determined using SPSS version 26.0 (IBM, Armonk, NY, USA); r^2^ was also computed in order to find out the cross-species inconsistency in RFC clarified by the discrepancy in UV following Amjad et al. [50].

### 2.5. Reverse Pharmacological Correlations

Eclectic screening of the relevant literature was carried out to review the pharmacology and phytochemistry of each documented plant species to validate these traditional ethno-veterinary medicines. Data were retrieved from Google Scholar, Science Direct, PubMed, Scopus, SciFinder and Web of Science by searching for the following keywords: phytochemicals, phytochemistry, biologically active compounds, biological activities, pharmacology, and medicinal uses in combination with each plant species.

## 3. Results

### 3.1. Demography of the Informants

Out of the total 59 informants, 64.40% were males and 35.59% were females. Based on age, the informants were divided into three age groups: 20–35 years (25.42%), 36–50 years (38.98%), and above 50 years (35.59%). Out of the total informants, 37 were married and 22 were unmarried. Concerning education, 32.20% were illiterate, 22.03% had attended school up to primary level, 16.95% up to middle level, 13.56% up to secondary level, 8.47% up to intermediate level, and 6.78% above higher secondary level (Table 1). The informants belong to two tribal groups: Gujjars (35 informants) and the Bakerwals (24 informants). The duration of time spent by the informants in the study area varies and it was found that Gujjars spend more time in the forest ecosystem for utilizing natural resources and either did not migrate or migrated within the district; Bakerwals are nomadic and migrate to other areas such as Kashmir valley during summer, and thus spend less time in the study area.

### 3.2. Medicinal Plant Diversity

During the present study, a total of 56 plant species belonging to 54 genera and 39 families were reported. The details of botanical names, vernacular names, families, life forms, ethno-veterinary uses, parts used, methods of use, FC, RFC, and UV are given in Table 2. Asteraceae with five species was dominant, followed by the Lamiaceae, Fabaceae, and Amaranthaceae with three species each. The remaining families contribute less than two species in the listed ethno-veterinary plants, out of which 28 families are represented by single species. The reason behind the dominance of Asteraceae, Lamiaceae, and Fabaceae in the traditional healthcare system may be due to the abundance of plant species of these families in the study area [53]. Residents of the study region have therefore been using these plants for many generations as a result of their easy accessibility, and as a result, they are familiar with the plant families. These plant families have been reported to be dominant in other ethnobotanical studies as well [54,55,56]. The reason for the overall dominance of the plant species belonging to these families may probably be due to the presence of secondary metabolites in them. Many active essential oils have been isolated from members of the family Lamiaceae [57]. Furthermore, plants of the family Asteraceae are known for their pharmacological importance [58,59,60] and this family is extensively strewn and is regarded as the largest angiosperm family in the world [61].

**Figure 3:**
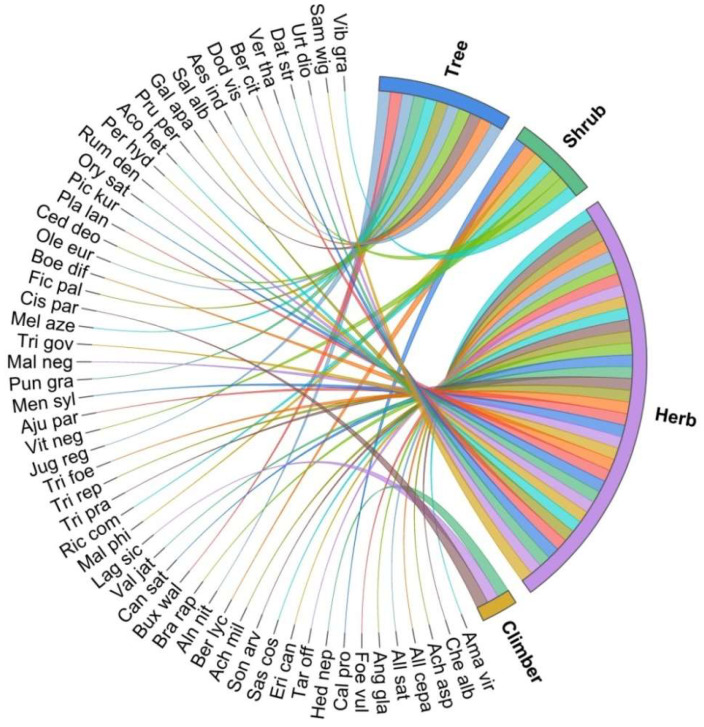
Growth form of the documented plant species.

Life forms are dominated by herbs, followed by trees, shrubs and climbers (Figure 3). In other similar studies [62,63,64], the life forms are dominated by herbaceous plants. The dominance of herbs in conventional and their use indigenous medicinal systems may be due to their easy availability, high therapeutic potential, and presence of biologically active phytochemicals [65,66,67]. Further, the presence of soft tissues in herbaceous plants makes their extractions and preparations uncomplicated and easy. Most of the documented plant species grow in the wild, and few (*Allium cepa*, *Allium sativum*, *Foeniculum vulgare*, *Brassica rapa*, *Lagenaria siceraria*, *Trigonella foenum-graecum*, *Mentha sylvestris*, *Oryza sativa*, and *Prunus persica*) are cultivated. The forests, farmlands, roadsides, fallow lands and riversides are the habitats where the wild medicinal plants are found.

### 3.3. Utilization Pattern

**Figure 4:**
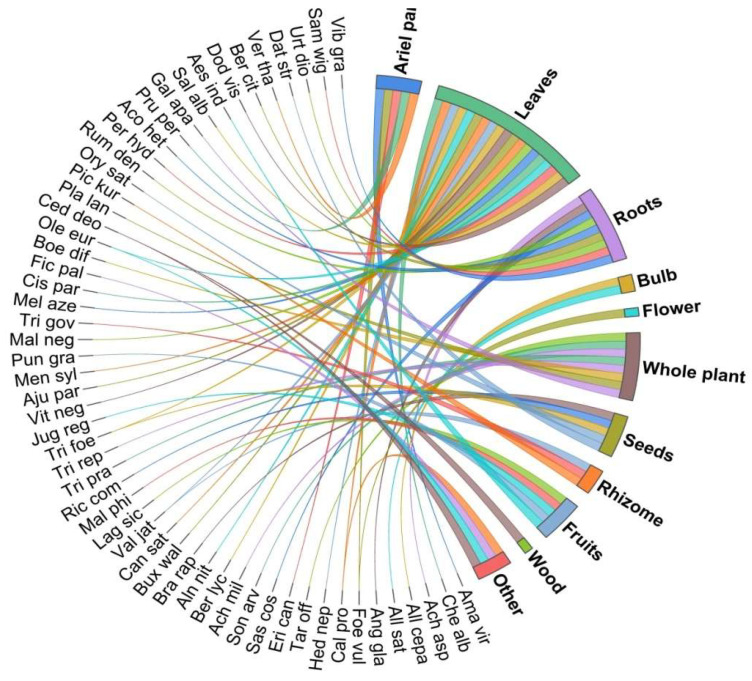
Utilization pattern of the documented plant species.

Depending on hereditary knowledge and the accessibility of those plants and plant parts to the local population, different plant parts, such as leaves, roots, stems, flowers, fruits, and even resins, gums, and galls of some plants, are used in diverse ways in traditional health care systems. In the present study, leaves were the most common plant parts used (35.71%), followed by roots (16.07%), whole plants (12.5%), fruits (10.71%), aerial parts (8.92%), seeds (7.14%), rhizome and bark (5.35% each), bulbs (3.57%), grains, and wood and latex (1.78%) (Figure 4). Leaves are often used in herbal preparations and have been documented as the most-used plant parts in many studies [68,69,70,71,72]. The reason for the preference of leaves in herbal medicines may be due to their easy collection [73] and the presence of bioactive metabolites such as alkaloids, flavonoids, terpenoids, saponins, phenolic compounds in leaves [74]. Further, the collection of leaves causes less damage to the plant [40]. Similarly, a high concentration of biologically active compounds in roots make them favored parts for curative uses, besides leaves [50].

### 3.4. Methods of Herbal Drug Preparations and Administration

**Figure 5:**
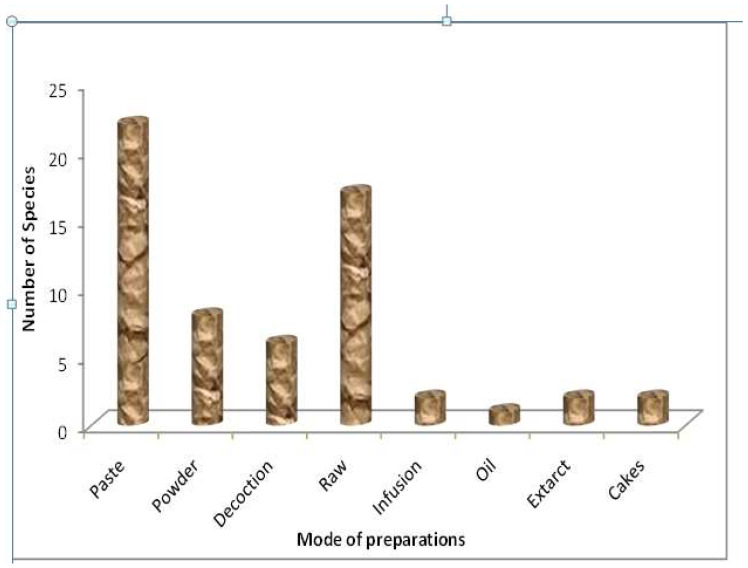
Mode of preparations of the documented plant species.

Herbal preparations were made through different modes i.e., infusion, poultice, powder, decoction, extract, juice, paste, tea, scorched, gel, cooked and steamed. In the present study, the main method of preparation was paste (36.6%), followed by raw (28.3%), powder (13.3%), decoction (11.1%), infusion, extract and cakes (3.33% each) and oil (1.6%) (Figure 5). Pastes are one of the common modes of preparation in ethno-medicinal practices due to their ease in preparations and handling. Furthermore, in pastes the originality and purity of plant material is maintained as no heat or any other treatment is used and thus does not alter the phytochemical composition of the plant material, leading to the acceleration of biological activities. Similar findings have been reported from some other studies also [75,76]. Preparations are applied both externally as well as internally. Dosages are determined on the basis of age, physical appearance health conditions, and the sternness of disease/ailment. Most of the plants are used directly but in some cases plant materials are mixed with additive materials and the amalgam is used as medicine. Common additives used include the husk of wheat and rice, salt, oil, flour and gur (raw sweet).

### 3.5. Quantitative Analysis

*Relative Frequency of Citation*: In the present study, the RFC value ranges from 0.27–0.81 (Table 2). The highest RFC was recorded for *Brassica rapa*, and the lowest RFC was recorded for *Picrorhiza kurroa* and *Trillium govanianum*. Some other species with a high RFC include *Taraxacum officinale* and *Trifolium pratense* (0.76 each), *Foeniculum vulgare* (0.73) and *Achillea millefolium* (0.71). The plant species with high RFC grow abundantly in the study area, so the local people are well aware of their therapeutic properties. Thus, among the indigenous people, their unique abilities to treat various illnesses and conditions have gained popularity. Such plant species should be evaluated for their phytochemical and pharmacological properties, as these plant species could lead to identifying bioactive compounds for novel drug discoveries [77].

*Use Value*: In the present study, UV ranges from 0.87 for *Brassica rapa* to 0.21 for *Trillium govanianum* (Table 2). Other species with UV high include *Trifolium pratense* (0.79) and *Taraxacum officinale* (0.78). The reason for the high UV of a species may be due to its easy availability, extensive distribution and high therapeutic properties for curing various diseases [78,79]. Plants having more use reports have higher use values, while those plants having fewer use reports have lower use values [50]. In the present study, the plant species having higher use values are common plants growing abundantly in the study area. Such plants with a higher UV suggest the presence of bioactive compounds and thus require thorough phytochemical evaluations [80].

*Informant Consensus Factor:* ICF is used to gauge how well the community agrees on the usage of several plant species for a given disease category. The ICF for the eight disease categories identified during the present study ranges from 0.93 to 0.97 (Table 3). The ICF for all disease categories was recorded as being high, revealing that the informants have the same opinion on which plants to use in the treatment of common diseases. The highest ICF was recorded for dermatological/wounds and jaundice/Foot and Mouth disease (0.97 each) followed by pregnancy and post-pregnancy (0.96), the musculoskeletal system, the gastrointestinal system and insecticide/antidote categories (0.95 each), pneumonia/cough/cold/fever (0.94) and hair loss, and ENT/ophthalmic system (0.93). The high ICF values signify a plausibly high consistency of informants on the uses of medicinal plant species [81]. For treating a single disease category, a high ICF value is frequently linked to a small number of specific plants with high use reports [82], whereas low values are linked to many plant species with comparable or high use reports, implying a lower level of agreement among the informants on the use of these plant species for treating a specific disease category [50].

*Jaccard Index:* The resemblances and disparities in ethno-medicinal studies seem to mark the significance of traditional medicinal knowledge in different regions, where historical, phytochemical and ecological factors interact in their selection [83,84]. Further, indigenous communities have differences in their origins and cultures leading to differences in ethnobotanical knowledge within these communities [85]. The results of regional, national, and international studies were compared with the data from the current study, and the observed percentage of similarity spans from 1.3 to 23.1 with an average value of 8.95 (Table 4). The maximum level of similarity was found with the studies conducted by Ch et al. [86], Sharma et al. [87], Khuroo et al. [88], and Khan et al. [89], with JI values of 23.1, 14.6, 11.9 and 10.5, respectively. It is interesting to note that all these studies were carried out in areas which are in close vicinity to our study area and thus have similar ecological factors and ethnic values. The lowest index of similarity was found in the study conducted by Harsha et al. [90] in Uttara Kannada district of Karnataka, and the reason for this may be the difference in topography, climate, vegetation cover and difference in socio-cultural values. According to JI, various tribal societies have their own traditional knowledge systems that employ identical plant bioresources in various ways. The great amount of information among groups can be revealed by recording and comparing ethnic/traditional knowledge, which can lead to new sources of drug discovery [83].

### 3.6. Statistical Analysis

**Figure 6:**
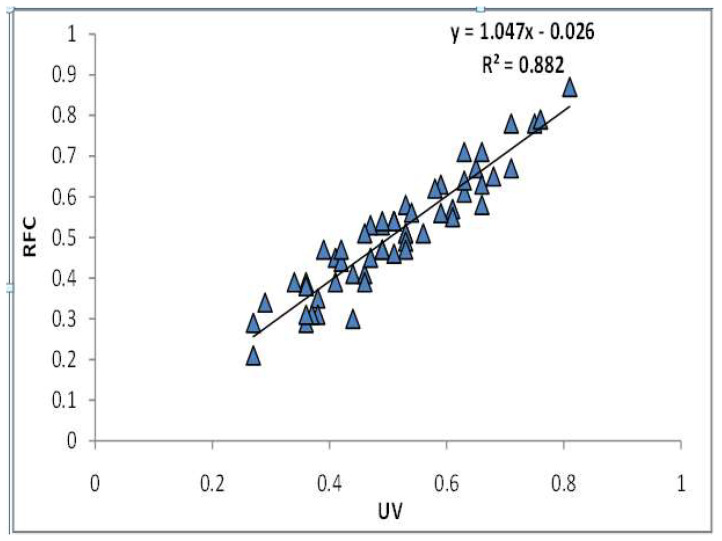
Relationship between RFC and UV.

The Pearson correlation coefficient between the use value and relative frequency of citations is 0.94, revealing a considerable and strong positive correlation between the proportion of uses of a plant species within a sample of respondents and the frequency with which a particular use of a species was mentioned by the informant (Table 5). This implies a direct relationship between the number of informants and use reports of a particular species. In the present study, R^2^ is 0.88, implying that 88% of the variation in RFC can be elucidated in terms of the UV [95,96], implying the empirical robustness among the two indices [66]. It also implies that Figure 6 illustrates the positive correlation between the values of RFC and UV.

### 3.7. Reverse Pharmacological Correlation of Ethno-Vetenairy Plants

Plant species documented during the present study have been found to show many biological activities due to the presence of various biologically active phytochemicals (Table 6). Some of the biological activities associated with these plants are antiviral, anticarcinogenic, spermicidal, hepatoprotective, nephroprotective, antidiabetic, anti-inflammatory, immunomodulatory, antimicrobial, antiparasitic, anti-allergic, antioxidant, hypolipidemic, antifungal, anthelminthic, antimicrobial, antidiabetic, and antipyretic. Prominent biologically active chemicals reported from the documented plants include quercetin, betaine, alliin, allicin, linalool, borneol, stigmasterol, lupeol, β-sitosterol, campesterol, rutin, saussurine, eugenol, limonene, α-thujone, artemetin, α-pinene, β-pinene, salicylic acid, berberine, berbamine, cannabigerol, cannabidiol, betulin, kaempferol, pennogenin, diosgenin, apigenin 7-glucoside, aconitine, heterophylline A, heterophylline B, and bergenin.

These phytochemicals are associated with several biological activities and thus play a vital role in healthcare systems. For example, allicin extracted from *Allium sativum* is a defensive phytochemical with a broad range of biological activities. It inhibits the proliferation of both bacteria and fungi, including antibiotic-resistant strains and furthermore, allicin induces cell-death and inhibits cell proliferation in mammalian cancer cells [219]. Similarly, berberine derived from *Berberis lycium* shows anti-inflammatory, antioxidant, anti-depressant and anti-hypertensive activities [220]. The biological activities of some other phytochemicals isolated from the documented plant species are given in Table 7.

On earth, there are approximately 250,000 plant species [234], and only 5–15% of higher plants have been thoroughly examined for the presence of bioactive compounds [235]. Plants have an immeasurable potential to produce a vast diversity of unusual chemical structures known as secondary metabolites that modulate the relationships of organisms with the environment [235]. Additionally, plants possess a precise mechanism to fight infection through the production of phytoalexins, possessing anti-infective activities. Such phytochemicals hold much potential for medicinal applications, and thus, it is coherent and reasonable to investigate the potential of such compounds for both human and animal health care. Studies of the biological activity of ethno-veterinary plants can provide clues about promising leads for the isolation and identification of useful compounds that may be developed into pharmaceuticals. The present study reported the use of *Chenopodium album* for wound healing by the local inhabitants, and Said et al. [236] have revealed that *C. album* can promote wound healing and tissue regeneration through the modulation of growth factors and their receptors. Similarly, *Foeniculum vulgare* was reported to cure gastrointestinal disorders and Birdane et al. [237] has revealed a protective effect of *F. vulgare* against ethanol-induced gastric mucosal lesions in rats. Thus, following up on ethno-medicinal leads is one of the best approaches to selecting plants for bioactivity screening. There is frequently an overlap between medicinal plants used to alleviate animals and humans. It would make sense that similar treatments are used to treat comparable ailments in humans and their livestock [238]. Ethno-veterinary medicines can function as leads for drug development, but probably a more practical and lucrative exercise would be to develop a preferred preparation through pharmacological research and development, and the ensuing medication can be returned to society with extra impact. Further, local farmers can cultivate such plants, which improves the economic condition of the farmers, and such commercialization can aid in biodiversity conservation. The growing interest in traditional medicine and increasing admiration of ethno-veterinary medicinal plants has been restricted in terms of more advancement by the inaccessibility of information on the effectiveness and assurance of these practices.

## 4. Conclusions

In the isolated rural areas of the Himalayan region, ethnobotanical knowledge is crucial for preserving plant diversity and treating a variety of diseases. One of the most crucial prevailing systems in the region where modern veterinary healthcare facilities are scarce or in extremely bad condition is the traditional method of treatment. The folk knowledge of ethno-veterinary medicine has its significance in the treatment of livestock diseases in remote and rural areas of the J&K. Farmers alleviate the health-related problems of their livestock using ethno-veterinary medicine, as it is affordable and a more reliable surrogate to synthetic drugs. The seminomadic and nomadic tribes of the Indian Himalayan region in general and the Gujjar and Bakerwal tribes of J&K, in particular, still possess a rich heritage of traditional healthcare systems. In our literature survey, it was found that plant species documented during the present study possess biological activities owing to the presence of various biologically active phytochemicals. Thus, more studies of the biological activities of ethno-veterinary plants can provide clues of promising leads for the isolation and identification of useful compounds that may be developed into pharmaceuticals. Further, there is recurrently an overlap between medicinal plants used to alleviate animals and humans. Thus, ethno-veterinary medicines can function as leads for drug development, but probably a more practical and lucrative exercise would be to develop a preferred preparation by pharmacological research, and the ensuing medication can be returned to the society with extra impact. However, this valuable knowledge-base has become obsolete due to industrialization, urbanization and, above all, lack of recognition by the younger generations. Therefore, there is a dire need to review, document and authenticate the valuable traditional knowledge of ethno-medicinal plants for the human welfare. This study could be a pilot to document the sustainable utilization of frequently used bioresources by understanding the traditional knowledge systems, and will provide important leads for the formulation of novel drugs and medicine.

## Figures and Tables

**Table 1:** Demographic data of informants.

S. No.	Variable	Category	No. of Informants	% Age
1	Gender	Male	38	64.40
		Female	21	35.59
2	Marital status	Married	37	62.7
		Unmarried	22	37.3
2	Age (in years)	20–35	15	25.42
		36–50	23	38.98
		Above 50	21	35.59
3	Educational Qualification	Illiterate	19	32.20
		Primary	13	22.03
		Middle	10	16.95
		Secondary	8	13.56
		Hr. Sec.	5	8.47
		Above	4	6.78

**Table 2:** Ethno-veterinary plants used by Gujjar and Bakerwal tribes in Poonch, J&K.

Taxa/Local Name	Family/ Voucher Number	Life Form	Ethno-Veterinary Uses	Part(s) Used	Preparation	Method of Use	FC	RFC	UV
*Amaranthus viridis* L. {Ganar}	Amaranthaceae/BGSBU-01	H	Tonic	AP	Raw	Aerial parts of the plant are cut into small pieces and mixed with wheat husk. This mixture is fed preferably twice a day for two weeks.	23	0.38	0.35
*Chenopodium album* L. {Bathua}	Amaranthaceae/BGSBU-02	H	Wound healing	L	Paste	Paste prepared from the leaves boiled in mustard oil is applied externally.	32	0.53	0.58
*Achyranthes aspera* L. {Phutkanda}	Amaranthaceae/BGSBU-03	H	Fever	R	Paste	Root paste is given orally.	39	0.65	0.68
			Poisonous bite	R	Infusion	Infusion of root is given to cattle.			
*Allium cepa* L. {Gandha}	Amaryllidaceae/ BGSBU-04	H	Loss of appetite	B	Paste	Paste of bulbs mixed with salt is fed to the cattle.	37	0.61	0.58
			Stimulate oestrus cycle	B	Paste	Crushed bulbs are mixed with salt and given to cows.			
			Skin infection	B	Paste	Paste prepared from the crushed bulbs is applied on the infected body part.			
*Allium sativum* L. {Thoom}	Amaryllidaceae/ BGSBU-05	H	Deworming	B	Paste	Paste of crushed bulbs is mixed with flour and given orally.	23	0.38	0.31
*Angelica glauca* Edgew. {Chora}	Apiaceae/BGSBU-06	H	Cold	R	Decoction	Decoction of root is given to cattle thrice a day.	31	0.51	0.54
			Diarrhea	R	Paste	Root paste is given to cattle.			
			Alopecia	R	Paste	Root paste is applied externally.			
*Foeniculum vulgare* Mill. {Sounf}	Apiaceae/BGSBU-07	H	Indigestion	AP	Decoction	Aerial parts are boiled in water and are fed to the animal for 2–3 days	43	0.71	0.78
			Constipation	F	Decoction	Decoction prepared by boiling fruits in water is given to cattle.			
*Calotropis procera* (Aiton) W.T.Aiton {Aak}	Apocynaceae/ BGSBU-08	S	Removal of retained placenta	Ltx	Raw	Tail of buffaloes is dipped for 4–5 min into latex.	38	0.63	0.61
			Deworming	L	Raw	Green leaves are given as feedstuff daily.			
			increase milk production	L	Raw	Dried leaves are given as feedstuff especially in case of goat to increase the milk quantity.			
*Hedera nepalensis* K.Koch {Harbembel}	Araliaceae/BGSBU-09	C	Leech removing	L	Extract	Leaf extract is put in the nostrils.	41	0.68	0.65
*Taraxacum officinale* F.H.Wigg. {Handh}	Asteraceae/BGSBU-10	H	Enhance milk production	WP	Raw	Whole plant is fed to cattle with other feeds.	45	0.75	0.78
			Stretch of bones and ligaments	AP	Decoction	Decoction prepared by boiling aerial parts into water in 1:1 ratio is given for about 15 days.			
*Erigeron canadensis* L. {Kuttey Haddi}	Asteraceae/BGSBU-11	H	Indigestion	AP	Paste	Aerial parts of the plants are crushed and the paste is fed to the cattle.	29	0.49	0.47
*Saussurea costus* (Falc.) Lipsch. {Kuth}	Asteraceae/BGSBU-12	H	Tonic	R	Powder	Root powder mixed with crushed onion bulbs, gur (raw sugar) and water is fed to the cattle.	21	0.36	0.38
*Sonchus arvensis* Linn. {Sonchal}	Asteraceae/BGSBU-13	H	Enhance milk production	WP	Raw	For increasing milk production, fresh plants are fed to cattle.	39	0.66	0.71
*Achillea millefolium* L. {Chou}	Asteraceae/BGSBU-14	H	Deworming	WP	Raw	Whole plants are fed to animals.	42	0.71	0.67
*Berberis lycium* Royle {Simboo}	Berberidaceae/ BGSBU-15	S	Wound healing	L	Paste	Leaves are chewed and this paste is applied on the wounds.	35	0.59	0.63
*Alnus nitida* (Spach) Endl. {Sarol}	Betulaceae/BGSBU-16	T	Foot and mouth disease	L	Paste	Dried leaves are mixed with oil and applied on the affected parts.	31	0.53	0.51
*Brassica rapa* L. {Sariyoon}	Brassicaceae/BGSBU-17	H	Enhance milk production	Sd	Cakes	Seed cakes locally called *Khahl* is fed to lactating cows and buffaloes to enhance milk production.	48	0.81	0.87
			Vigor maintenance of bulls	Sd	Cakes	Mixture of seed cakes and rice husk is fed to bulls.			
			Skin Infection	Sd	Paste	Seeds are ground and mixed with mustard oil. This paste is applied externally on infected parts for a week.			
*Buxus wallichiana* Baill. {Chikhri}	Buxaceae/BGSBU-18	T	Skin infection	L	Decoction	Decoction of fresh or dried leaves is given orally.	24	0.41	0.45
*Cannabis sativa* L. {Bhang}	Cannabaceae/ BGSBU-19	H	Removal of lice and ticks	L	Paste	Paste of crushed leaves is applied externally.	31	0.53	0.51
*Valeriana jatamansi* Jones ex Roxb. {Balo}	Caprifoliaceae/ BGSBU-20	H	Fatigue	Rh	Powder	Dried rhizomes are ground to fine powder which is dissolved in about 200–300 mL of normal water and is given to the cattle in the morning for about a week.	29	0.49	0.53
			Diarrhea	L	Raw	Fresh leaves are used directly or their extract to cure diarrhea.			
*Lagenaria siceraria* (Molina) Standl. {Doberi}	Cucurbitaceae/ BGSBU-21	C	Yoke Galls	F	Paste	Fruits are burned. Ash is mixed with luke warm mustard oil and the paste is applied on yoke galls of bulls.	22	0.37	0.31
*Mallotus philippensis* (Lam.) Mull. Arg. {Kamila}	Euphorbiaceae/ BGSBU-22	T	Deworming	F	Powder	Powdered dry fruits are mixed with flour and given to animals for 2–3 days.	29	0.49	0.47
*Ricinus communis* L. {Arand}	Euphorbiaceae/ BGSBU-23	S	Dysentery	Sd	Paste	Seeds are crushed in small quantity, mixed with fodder and given to cattle.	30	0.51	0.53
*Trifolium pratense* L. {Shatul}	Fabaceae/BGSBU-24	H	Enhance milk production	WP	Raw	Whole plant is fed to cattle	45	0.76	0.79
*Trifolium repens* L. {Srieh}	Fabaceae/BGSBU-25	H	Enhance milk production	WP	Raw	Whole plant is fed to cattle	39	0.66	0.63
*Trigonella foenum-graecum* L. {Methi}	Fabaceae/BGSBU-26	H	Diarrhea	L Sd	Raw	Leaves and seeds are fed to animal for 3–4 days.	34	0.58	0.62
*Juglans regia* Linn. {Khor}	Juglandaceae/ BGSBU-27	T	Enhancing Milk Production	F	Cakes	The oil cakes obtained by grinding of fruit kernels are fed to cows to enhance their milk production.	26	0.44	0.40
*Vitex negundo* L. {Banno}	Lamiaceae/BGSBU-28	S	Fever	L	Paste	Young leaves are crushed and given orally.	24	0.41	0.38
*Ajuga parviflora* Benth. {Jan-i-adam}	Lamiaceae/BGSBU-29	H	Weakness	L	Infusion	Water extract of fresh leaves is given to cattle	30	0.51	0.53
			Indigestion	L	Decoction	Decoction is given to animals orally			
			Fever	L	Decoction	Decoction is given to animals orally			
*Mentha sylvestris* L. {Pootno}	Lamiaceae/BGSBU-30	H	Deworming	L	Raw	Leaves are fed to live stock	27	0.46	0.41
*Punica granatum* L. {Daruno}	Lythraceae/BGSBU-31	T	Jaundice	F	Decoction	Decoction of fruit exocarp is given orally.	37	0.63	0.71
*Malva neglecta* Wall. {Sonchal}	Malvaceae/BGSBU-32	H	Constipation	L	Paste	Soaked leaves are crushed, mixed with cow-butter and fed to newly born calves.	28	0.47	0.53
			Detachment of placenta in Cows	L	Paste	Paste is fed to cows to facilitate the detachment and expulsion of placenta after delivery.			
*Trillium govanianum* Wall. ex D.Don {Trae patri}	Melanthiaceae/ BGSBU-33	H	Deworming	Rh	Paste	Crushed rhizome is given to the cattle.	16	0.27	0.21
*Melia azedarach* L. {Dreck}	Meliaceae/BGSBU-34	T	Foot and mouth disease	L	Paste	Fresh leaves are crushed with sugar and water. The paste so formed is given orally to the cattle for 2–3 days.	31	0.53	0.48
*Cissampelos pareira* L.	Menispermaceae/ BGSBU-35	C	Eye problems	L	Infusion	Infusion of leaves is put in eyes	21	0.36	0.38
*Ficus palmata* Forssk. {Kemeri}	Moraceae/BGSBU-36	T	Wounds	BK	Raw	Bark is applied on wound for quick healing.	36	0.61	0.55
			Fracture	Bk	Raw	Bark is wrapped around broken bones.			
*Boerhavia diffusa* L. {Itt-sitt}	Nyctaginaceae/ BGSBU-37	H	Removal of retained placenta	WP	Raw	Whole plants are fed twice a day.	28	0.47	0.45
*Olea europaea* Subsp. cuspidata (Wall. ex. G. Don) Cif. {Kaou}	Oleaceae/BGSBU-38	T	Deworming	L	Raw	Fresh leaves are given for deworming.	39	0.66	0.58
			Fracture	Bk	Raw	Fresh stem bark is tied over broken bones.			
*Cedrus deodara* (Roxb. ex D. Don.) Don. {Dyar}	Pinaceae/BGSBU-39	T	Vomiting	W	Oil	Small pieces of wood are heated in a vessel which causes an oil to ooze out from them. This oil is given to live stock in vomiting.	27	0.46	0.51
			Hair fall in Goats	W	Oil	Oil is also applied externally to cure hair fall in goats.			
			Removal of ticks and lice	W	Oil	Oil is also applied externally.			
*Plantago lanceolata* Linn. {Chamch-e-pater}	Plantaginaceae/ BGSBU-40	H	Yoke galls	WP	Paste	Paste is applied externally.	29	0.49	0.54
*Picrorhiza kurroa* Royle. ex Benth. {Koudh}	Plantaginaceae/ BGSBU-41	H	Pneumonia	Rh	Powder	Dried rhizome powder mixed with wheat flour, gur and water is fed to cattle.	16	0.27	0.29
			Tapeworm	Rh	Paste	Paste is given orally.			
*Oryza sativa* Linn. {Chaval}	Poaceae/BGSBU-42	H	Detachment and expulsion of placenta	Sd	Raw	Grains are fed to cows after delivery to facilitate the detachment and expulsion of placenta.	35	0.59	0.56
			Constipation	Sd	Paste	Paste of rice flour is made which is given to sheep.			
*Rumex dentatus* L. {Hullo}	Polygonaceae/ BGSBU-43	H	Gaseous bloats	R	Paste	Fresh roots are crushed, salt is added and small balls are made which are given to cattle.	33	0.56	0.51
			Cough	R	Paste	Paste is fed to cattle.			
			Sprained body parts	R	Paste	Paste is mixed with salt and is given orally.			
*Persicaria hydropiper* L.) Delarbre {Pipla}	Polygonaceae/ BGSBU-44	H	Tongue infection	L	Raw	Chopped leaves are applied on the tongue.	25	0.42	0.44
*Aconitum heterophyllum* Wallich ex Royle {Patris}	Ranunculaceae/ BGSBU-45	H	Flatulence	R	Powder	Root powder is given with water.	17	0.29	0.34
*Prunus persica* (Linn.)Batsch.{Aarou}	Rosaceae/BGSBU-46	T	Wound healing	L	Paste	Paste is applied externally.	25	0.42	0.47
*Galium aparine* L.	Rubiaceae/BGSBU-47	H	Wound healing	WP	Paste	Paste is applied externally.	21	0.36	0.29
*Salix alba* L. {Beeso}	Salicaceae/BGSBU-48	T	Deworming	Bk L	Decoction	Bark decoction and leaves are given to animals.	32	0.54	0.56
*Aesculus indica* (Wall. ex Camb.)Hook. {Bankhori}	Sapindaceae/BGSBU-49	T	General weakness	F	Paste	Crushed fruits mixed with onion and salt are fed to cattle.	31	0.53	0.47
*Dodonaea viscosa* Jacquin {Sanatha}	Sapindaceae/BGSBU-50	S	Deworming	L	Extract	Leaf extract is given to cattle.	20	0.34	0.39
*Bergenia ciliata* (Haw.) Sternb. {Bat mevo}	Saxifragaceae/ BGSBU-51	H	Diarrhoea	R	Powder	Dried roots are powdered which is given to cattle with luke-warm water.	30	0.51	0.46
			Enhance milk production		Paste	Paste is fed to the cattle.			
*Verbascum thapsus* L. {Gidharh tamako}	Scrophulariaceae/ BGSBU-52	H	Flatulence	AP	Decoction	Decoction is prepared by boiling aerial parts in water for about 2 h, is added to the paddy husk and given to the cattle to cure flatulence.	26	0.44	0.3
*Datura stramonium* L. {Daturo}	Solanaceae/BGSBU-53	H	Leech removing	Sd	Raw	Dried seeds are heated on fire to release smoke which is used to expel leeches from the nasal cavity.	21	0.36	0.31
*Urtica dioica* L. {Kiyarie}	Urticaceae/BGSBU-54	H	Fractured bones	R	Paste	Root paste is applied on the fractured bones for early healing.	23	0.39	0.47
*Sambucus wightiana* Wall.	Viburnaceae/BGSBU-55	H	Foot and mouth disease	R	Paste	Paste is applied externally.	27	0.46	0.39
*Viburnum grandiiflorum* Wall ex. Wt & Arn. {Kuch}	Viburnaceae/BGSBU-56	S	Wound healing	R	Powder	Powdered roots mixed with mustard oil is applied externally.	37	0.63	0.64

*Abbreviations used:* H = Herb; S = Shrub; T = Tree; C = Climber; R = Root; F = Fruit; L = Leaf; B = Bulb; Sd = Seed; W = Wood; AP= Aerial parts; WP= Whole plant; Rh = Rhizome; Ltx = Latex; Bk = Bark.

**Table 3:** Information Consensus Factor or Participatory Agreement Ratio of informants.

Disease Category	Ailments Included	N_ur_	N_t_	ICF
Musculoskeltal System	Body weakness, Stretch of bones and ligaments, fatigue, Fracture, sprains	196	10	0.95
Pneumonia/Cough/Cold/Fever	Pneumonia, Cough, Cold, Fever	88	6	0.94
Dermatological/wounds	Skin diseases, wounds, yolk galls	268	9	0.97
Alopecia/Ophthalmic	Hair loss, eye disease	47	3	0.93
Gastrointestinal System	Indigestion, vomiting, diarrhea, dysentery, flatulence, tongue infection, gaseous bloats, constipation, loss of appetite	315	16	0.95
Jaundice/Foot and Mouth disease	Jaundice, Foot and Mouth disease	126	4	0.97
Pregnancy and post pregnancy	Stimulate oestrus cycle, Removal of retained placenta, increased milk production, Vigor maintenance	322	12	0.96
Insecticide/Antidote	Poisonous bite, deworming, removing of lice and ticks, removing of leaches	345	15	0.95

**Table 4:** Jaccard index comparing the present study with some of the earlier reports.

Area	Study Year	No. of Recorded Plant Species	Plants with Similar Use	Plants with Dissimilar Use	Total Species Common in Both Areas	Species Enlisted Only in Aligned Area	Species Enlisted Only in Study Area	% of Plants with Similar Use	% of Plants with Dissimilar Use	Jaccard Index	Citation
Kashmir Himalaya	2007	24	5	2	7	17	49	20.8	8.3	11.9	[88]
Kathua district of J&K	2012	72	5	8	13	59	43	6.9	11.1	14.6	[87]
Samahni valley, district Bhimber (Azad Kashmir) Pakistan	2006	54	9	6	15	39	41	16.7	11.1	23.1	[86]
Poonch valley, Azad Kashmir	2012	19	3	3	6	13	50	15.8	15.8	10.5	[89]
Tosham block of district Bhiwani (Haayana)	2014	52	3	2	5	47	51	5.8	3.8	5.4	[91]
Jhansi district, UP	2010	47	0	7	7	40	49	0	14.9	8.5	[92]
Tehri district of Garwal Himalaya	2013	35	0	5	5	30	51	0	14.3	6.6	[93]
Uttara Kannada district of Karnataka	2005	24	0	1	1	23	55	0	4.2	1.3	[90]
Visakhapatnam and Vizianagarm districts, AP	2015	61	0	3	3	58	53	0	4.9	2.8	[94]
Kudavasal taluk of Thiruvarur district, TN	2016	54	0	6	6	48	50	0	11.1	6.5	[12]

**Table 5:** Correlation between RFC and UV.

Correlation between RFC and UV		RFC	UV
RFC	Pearson Correlation	1	0.940 **
	Sig. (2-tailed)		0.000
	N	56	56
UV	Pearson Correlation	0.940 **	1
	Sig. (2-tailed)	0.000	
	N	56	56

** Correlation is significant at the 0.01 level (2-tailed).

**Table 6:** Reverse pharmacological correlations of documented ethno-veterinary plants from Poonch district of J&K.

Taxa	Phytochemicals	Pharmacological Activities	Reference(s)
*Amaranthus viridis*	rutin, quercetin, spinosterol, amasterol	antifungal, hepatoprotective, anthelminthic, antioxidant, antimicrobial, antidiabetic, antipyretic, anti-inflammatory	[97,98,99,100]
*Chenopodium album*	3-O-glycosides of caempferol, quercetin, andisoramnetin, kaempferol-3-O-(4-β-D-xylopyranosyl)-α-L-rhamnopyranoside-7-O-α-L-rhamno-pyranoside,3-O-(4-β-D-apiofuranosyl)-α-L- rhamnopyranoside-7-O-α-L rhamnopyranoside,3,7-di-O-α-L-rhamnopyranoside, 3-O-glucopyranoside, quercetin 3,7-di-O-β-D-glucopyranoside, 3-O-glucosylglucuronide, 3-O-α-L-rhamnopyranosyl-(1→6)-β-D-glucopyranoside,3-O-β-D-glucopyranoside, kaempferol-3-O-arabinoglucoside, quercetin, quercetin 3-O-xylosylglucoside, quercetin-3-orhamnoglucoside	antiviral, antifungal, anti-inflammatory, antiallergic, antiseptic, antipruritic, anti-nociceptic, sperm immobilizing immunomodulating, antiparasitic, antispasmodic, antibacterial, anti-helminthic, hypotensive, spasmolytic, hepatoprotective	[101,102,103]
*Achyranthes aspera*	betaine, achyranthine, hentriacontane, ecdysterone, achyranthes saponins A, B, C, D, α-Lrhamnopyranosyl-(1→4)-(β-Dglucopyranosuluronic acid)-(1→3)-oleanolic acid, trigmasta-5, 2-dien-3-β-ol, trans-13-docasenoic acid, n-hexacosanyl-n-decaniate, hexacos-17-enoic acid	antiviral, anticarcinogenic, spermicidal, hepatoprotective, nephroprotective, antidiabetic, anti-inflammatory, immunomodulatory, antimicrobial, antiparasitic, anti-allergic, anti-oxidant, hypolipidemic	[104,105,106]
*Allium cepa*	quercetin 3,7,4′-*O*-*β*-triglucopyranoside, quercetin 3,4′-*O*-*β*-diglucopyranoside, taxifolin 4′-*O*-*β*-glucopyranoiside	analgesic, antidiabetic, antioxidant, antidepressant, aphrodisiac, antihyperlipidemic	[107,108,109]
*Allium sativum*	alliin, allicin, ajoenes, vinyldithiins, quercetin	anticarcinogenic, antioxidant, antidiabetic, reno-protective, anti-atherosclerotic, antibacterial, antifungal, antihypertensive, antiviral, antifungal, antiprotozoal, antioxidant, anti-inflammatory, and anticancer	[110,111,112]
*Angelica glauca*	aleric acid, angelic acid, angelisine, phellandrene, coumarins, bergapten, linalool, borneol, anthotoxin, umbelliferene	cardioactive, carminative, digestive, sudorific, expectorant and stomachic, antipsoriatic, anti-bacterial, antifungal	[113,114,115,116,117]
*Foeniculum vulgare*	*trans*-anethole, fenchone, methylchavicol, eriodictyol-7-rutinoside, quercetin-3-rutinoside, rosmarinic acid, quercetin-3-glucuronide, isoquercitrin, quercetin-3-arabinoside, kaempferol-3-glucuronide, kaempferol-3-arabinoside, isorhamnetin glucoside, 3-*O*-caffeoylquinic acid, 4-*O*-caffeoylquinic acid, 5-*O*-caffeoylquinic acid, 1,3-*O*-di-caffeoylquinic acid, 1,4-*O*-di-caffeoylquinic acid, 1,5-*O*-di-caffeoylquinic acid	antimicrobial, antiviral, anti-inflammatory, antimutagenic, antinociceptive, antipyretic, antispasmodic, antithrombotic, apoptotic, cardiovascular, chemomodulatory, antitumor, hepatoprotective, hypoglycemic, hypolipidemic, and memory enhancing property	[118]
*Calotropis procera*	voruscharin, uscharidin, uzarigenin, calotroposide, calactin, calotoxin, uscharin, ascleposide, calotropagenin, coroglaucigenin, calotropin, proceroside, proceragenin, syriogenin, rutin, cyaindin-3-rhamnoglucoside, cycloart-23-en-3β, 25-diol, cyclosadol, multiflorenol, procestrol, quercetin-3- rutinoside, β-sitosterol, β-sitost-4en-3one, stigmasterol, cyanidin-3-rhamnoglucose, ascorbic acid, calactin, calotoxin, calatropagenin, calotropin, polysaccharide containing D-arabinose, D-glucose, D-glucosamine L-rhamnose, calotropagenin, 3-proteinase, calotropin, α-calotropeol, 3-epimoretenol, gigantin, giganteol, isogiganteol, α-lactuceryl acetate, α-lactuceryl isovalerate, lupeol, proceroside, proceragenin, syriogenin, taraxast-20α- (30)-en-(4-methyl-3-pentenoate), 3′-thiazoline cardenolide uscharidin, uzarigenin, voruscharin, β-sitosterol, α- and β-amyrin, lupeol, taraxasteryl acetate, α-and β-calotropeol, 3-epimoretenol, multiflorenol, cyclosadol, several triterpene esters, β-sitosterol, stigmasterol, calotropin, procerain, procerain-B, calactinic acid, choline, O-pyrocatechuic acid, β-sitosterol, taraxasterol, calotroprocerol A, calotroproceryl acetate A, calotroprocerone A, calotroproceryl acetate B	analgesic, antinociceptive, anticonvulsant, antimalarial, anthelminthic, antioxidant, antidiabetic, anticancer, antimicrobial, anti-inflammatory, immunomodulatory, antipyretic, antidiarrheal, antidematogenic, antiplasmodial	[119,120,121,122]
*Hedera nepalensis*	lupeol, hederacoside C, α-hederin	anticancer, neuroprotective, antidiabetic, antioxidant	[123,124]
*Taraxacum officinale*	stigmasterol, campesterol, syringing, dihydrosyringin, dihydroconiferin, luteolin 7 glucoside, luteolin 7 diglucosides, cichoriin, aesculin, dicaffeoyltartaric acid, rutin, hiperoside, quercetin	antibacterial, antioxidant, anticancer, diuretic, hepatoprotective, antiviral, anti-inflammatory	[125,126,127,128,129]
*Erigeron canadensis*	onyzolide, conyzapyranone A, conyzapyranone B, 4 Z,8 Z-matricaria-γ-lactone, 4 E,8 Z-matricaria-γ-lactone, 9,12,13-trihydroxy-10(E)-octadecenoic acid, epifriedelanol, friedeline, taraxerol, simiarenol, spinasterol, stigmasterol, β-sitosterol, quercetin-7-O-beta-D-galacto pyranoside, quercetin, luteolin, apigenin, 5,7,4’-trihydroxy-3’-methoxy flavone, quercetin-3-alpha-rhamnopyranoside, quercetin-3-O-beta-D-glucopyranoside, apigenin-7-O-beta-D-gluco pyranoside, luteolin-7-O-beta-D-glucuronide methyl ester,4’-hydroxy baicalein-7-O-beta-D-glucopyranoside, baicalein, rutin	antimicrobial, antioxidant, anticoagulant, anti-inflammatory, anticancer, antifungal	[130,131,132]
*Saussurea costus*	costunolide, dehydrocostuslactone, costic, palmitic, linoleic acids, cyclocostunolide, alantolactone, isoalantolactone, isodehydrocostus lactone, iso-zaluzanin-C, guiainolides, 12-methoxydihydrodehydrocostuslactone, 4-methoxydehydrocostus lactone, saussurealdehyde, isodehydrocostus-lactone-15-aldehyde, cynaropicrin, reynosin, santamarine, Saussureal, pregnenolone, sitosterol, daucosterol, syrine, chlorogenic acid, saussurine	anti-inflammatory, anticancer, hepatoprotective, immunomodulatory, anti-ulcer, antimicrobial, hypoglycemic, antiparasitic	[133,134,135,136,137,138]
*Sonchus arvensis*	alkaloids, flavonoids, phenols, saponins and tannins	anti-fatigue activity, antioxidant, hepatoprotective, kidney-protective, antidiabetic, antibacterial	[139,140,141]
*Achillea millefolium*	borneol, camphene, azulene, carophyllene, 1,8-cineole, p-cymene, eugenol, farnesene, limonene, myrcene, α-pinene, β-pinene, salicylic acid, achillicin, achillin, terpinolene, α-thujone, artemetin, casticin, isorhamnetin, luteolin, rutin	antibacterial, antifungal, antiparasitic, hemostyptic, anti-inflammatory, antispasmodic, antioxidant, anticancer, hepatoprotective,	[142,143,144]
*Berberis lycium*	berberine, berbamine, chinabine, karakoramine, palmatine, balauchistanamine, gilgitine, jhelumine, punjabine, sindamine, chinabine acetic acid, maleic acid, ascorbic acid, baberine, berbericine hydrochloride, berbericine hydroiodide, oxyberberine, umbellatine	antidiabetic, hepatoprotective, anti-hyperlipidemic, antimutagenic, antioxidant, antidiarrheal, anti-arrhythmic, anti-depressant, anti-microbial, anti-protozoal	[145,146]
*Alnus nitida*	Diarylheptanoids	anticancer, anti-inflammatory, anti-influenza, hepatoprotective, antitumor and anti-oxidant	[147,148,149]
*Brassica rapa*	lutein, *β*-carotene, glucobrassicin, 4-methoxyglucobrassicin, 1-methoxyglucobrassicin, isothiocyanates, nitriles, thiocyanates, epithionitriles, oxazolidine, aconitic, citric, ketoglutaric, malic, shikimic, fumaric, oxalic, ascorbic, succinic and glutamic acids	anticancer, antioxidant, anti-inflammatory, chemopreventive	[150,151]
*Buxus wallichiana*	buxemenol E, buxaltine H, Buxiramin D, buxatinebuxandrine F, buxidine F, (+)-16a, 31-diacetylbuxadine, semperviraminol, buxamine F	bitter tonic, diaphoretic, anti-rheumatic, vermifuge, anti-helminthic, analgesic, purgative diuretic, antiepileptic, antileprotic, hemorrhoids	[152,153,154]
*Cannabis sativa*	cannabigerol, cannabichromene, cannabidiol, tetrahydrocannabinol, 9-tetrahydrocannabivarin, annabicyclol, annabinol, D-limonene, beta-myrcene, alpha-pinene, caryophyllene oxide, D-linalool, beta-caryophyllene	anticonvulsant, antibiotic, antifungal, anti-inflammatory, analgesic, anxiolytic, antipsychotic, antioxidant, antispasmodic, anti-emetic, sedative	[155]
*Valeriana jatamansi*	baldrinal, homobaldrinal, decyl baldrinal, valtroxal, isovalepotriate, acetoxyvalepotriate, isovalemxyhydroxy-dihydrovatrate, rupesin, linarin, linarin-isovalerianate, linarin-2-O-methylbutyrate, hispidulin, hesperetin-7-O-β-rutinoside, hesperidin, kaempferol 3-O-β-D-glucopyranoside, quercetin 3-O-β-D-glucopyranoside, kaempferol, quercetin, 7-O-β-D-glucopyranoside, apigenin 7-O-β-D- glucopyranoside, lariciresinol,, pinsepiol, syringaresinol, pinoresinol, berchemol, podophyllotoxin, hydroxyvalerenic acid, acetoxyvalerenic acid, valerenic acid	neuroprotective, sedative, cytotoxic, cardio-protective, anxiolytic, antidepressant, anti-inflammatory, antispasmolytic, hepatoprotective, antioxidant	[156]
*Lagenaria siceraria*	β-carotene, 22-deoxocurcubitacin-d, 22-deoxoisocurcubitacin d, avenasterol, codisterol, elesterol, isofucasterol, stigmasterol, sitosterol, compesterol, spinasterol, 7-0-glucosyl-6-C-glucoside apigenin, 6-C-glucoside apigenin, 6-C-glucoside luteolin, 7,4′-O-diglucosyl- 6-C-glucoside apigenin	analgesic, anti-inflammatory, antihyperlipidemic, diuretic, hepatoprotective, anthelminthic, antibacterial, immunomodulatory, antistress, hepatoprotective, antioxidant	[157]
*Mallotus philippensis*	rottlerin, mallotoxin, iso rottlerin, crotoxigenin, betulin, friedelin, kamaladiol-3-acetate, lipeol, tannic acid, 3-hydroxy-D-A-friedoolean-3-en-2-one, 2β-hydroxy-D-A-friedooleanan-3-one, 3α-hydroxy-D-A-friedooleanan-2-one, betulin-3 acetate lupeol acetate, berginin acetylaleuritote acid, sitosterol, crotoxigenin, coroghcignin, homorottlerin	antioxidant, antimicrobial, anticancer, anti-viral, anti-inflammatory, hepatoprotective, anti-helminthic, analgesic	[158,159]
*Ricinus communis*	kaempferol-3-O-β-D-xyylopyranoside, Kaempferol-3-O-β-rutinoside, Kaempferol-3-O-β-D-glucopy-ranoside, Quercetin, Ricin, Rutin, Ellagic acid, Epicatechin, Fucosterol, Stigmasterol, α-pinene, 1,8-pinene, 1,8-cineole, β-caryophyllene, Ricinine, demetilricinine, Triricinolein, Lupeol	abortifacient, analgesic, antiasthmatic, anti-fertility, contraceptive, antidiabetic	[160]
*Trifolium pratense*	biochanin A, benzaldehyde, (Z)-β-caryophyllene, 3-methyl-1-butanol, 3-octanone, Z)-β-caryophyllene, β-farnesene, 6,10,14-trimethyl-pentadecanone, 3-methylbutyl butanoate, 3-methyl-1-butanol,3-methylbutanoic acid, ethyl-2-methylpentanoate	estrogenic, antiproliferative, antioxidant	[161,162]
*Trifolium repens*	4′,5,6,7,8-pentahydroxy-3-methoxyflavone and 5,6,7,8-tetrahydroxy-3-methoxyflavone, 3,7-dihydroxy-4′-methoxyflavone, 5,6,7,8-tetrahydroxy-4′-methoxyflavone, 3,5,6,7,8-pentehydroxy-4′-methoxyflavone, 6-hydroxy-kaempferol, 4′,5,6,7,8-pentahydroxyflavone, 3,4′-dimethoxykaempferol, quercetin and kaempferol, chalcone, chalcanol glucosides, repensin A and repensin B, gallocatechin, epigallocate-chin, gallocatechin-(4α-8)-epigallocatechin	antioxidant, antifungal, anticancer, antiaging, hepatoprotective, anti-inflammatory, antidiabetic	[163]
*Trigonella foenum-graecum*	disogenin, gitogenin, neogitogenin, homorientin saponaretin, neogigogenin, trigogenin, trigonelline and choline	immunomodulatory, antioxidant, chemo preventive, anticancer, antidiabetic, gastro protective, anti-inflammatory, antipyretic, anthelmintic, antigenotoxic, anti-plasmodial, hypocholesterolemic, antiseptic, aphrodisiac, astringent, bitter, demulcent, emollient, expectorant	[164,165]
*Juglans regia*	quercetin, and caffeic acid, paracomaric acid, juglone, ascorbic acid, quercetin arabinoside, quercetin xyloside and quercetin rhamnoside, Glutelins, globulins, albumin and prolamins, glansrins A, B and C, casuarinin, stenophyllarin	antioxidant, antidiabetic, antimicrobial, and hepatoprotective, antifungal, anti-hypertensive, renal protective, anti-inflammatory, antinociceptive, anticancer	[166,167]
*Vitex negundo*	friedelin, casticin, artemetin, terpinen-4-ol, α-terpineol, sabenine, globulol, spathulenol, β-farnesene, farnesol, α-pinene, β-pinene, linalool, terpinyl acetate, caryophyllene epoxide, caryophyllenol, vitexicarpin, viridiflorol, 5-odesmethylnobiletin, gardenin A, gardenin corymbosin, terpinen-4-ol, α-copaene, β-caryophyllene, β-elemene, camphene, α-thujene, α-pinene, sebinene, α-elemene, δ-elemene, β-elemene, β-eudesmol, camphene, careen, 1,8-cineol, α-phellendrene, β-phellendrene, α-guaiene, neral, geranial, bornyl acetate, nerolidol, β-bisabolol, cedrol, agnuside, lagundinin, viridiflorol, betulinic acid, ursolic acid, dimethoxyflavonone, vitexoside, agnuside, R-dalbergiphenol, negundin A, negundin B, vitexin cafeate, epifriedelinol	anxiolytic, analgesic, anti-inflammatory, neuroprotective, antibacterial, antipyretic, anticancer, antioxidant	[168,169]
*Ajuga parviflora*	ajugin A, ajugin B	antidiabetic, antioxidant, antibacterial	[170,171,172]
*Mentha sylvestris*	menthol, menthone, isomenthone, limonene, neomenthol, methyl acetate, beta-caryopyllene, piperitone, alpha- and beta-pipene, acacetin, chrysoeriol, diosmin, eriocitrin (eriodictoyl-7-o-rutinoside), hesperidin, hesperidoside, isorhoifolin, linarin, luteolin, menthoside, methyl rosmarinate, rutin, tilianine, narirutin, nodifloretin, caffeic acid, lithospermic acid, rosmarinic acid, protocatechuic acid, protocatechuic aldehyde, phytosterols, β-sitosterol, daucosterol; aloe-emodin, chrysophanol, emodin	carminative, stimulant, stomachic, aromatic, antiseptic, antispasmodic, sudorific, emmenagogue, anesthetic, anodyne	[173,174,175]
*Punica granatum*	punicalin, punicalagin, gallic acid, ellagic acid, cyaniding, delphinidin, uteolin, quercetin, kaempferol, naringenin, estrone, estriol, testosterone, betasistosterol, coumesterol, gammatocopherol, punicie acid, campesterol, stigmasterol	antioxidant, antibacterial, anticancer, anti-inflammatory, anticoagulant, antimutant, cardioprotective, antifungal, antidiabetic	[176,177,178]
*Malva neglecta*	hydrotyrosol, coumaroylhexoside, kaempferol-3-(p-coumaroyldiglucoside)-7-glucoside, quercetin-3-O-rutinoside, epicatechin-3-O-(4-O-methyl)-gallate, oleic acid, taurine, ethylene dimercaptan, isoeugenol, patchoulane, methyl 12-methyltetradecanoate, isopropyl myristate	antimicrobial, antioxidant, anti-inflammatory, anti-ulcerogenic, hepatoprotective, neuroprotective	[179,180,181,182]
*Trillium govanianum*	pennogenin, diosgenin, borassoside E, govanoside A	analgesic, anti-inflammatory, antifungal, antioxidant, anticancer, antispasmodic, diuretic	[183]
*Melia azedarach*	surinol, melianin b, sendanolactone, 3-α-hydroxy-4, 4,14alpha-trimethyl-5-αpreg-8-en-20-one, ochinin acetate, 4-methoxy-1-vinyl-beta-carboline, 4,8-dimethoxy-1-vinyl-beta-carboline, kuline, kulactone, kulolactone, kulinone, nimbinene, azaridine, paraisine, isochuanliansu, 6 H-β-hydroxy-4-stigmasten-3-one, 6βhydroxy-4-campesten-3-one, quercetrin, quercetin-3-0-β-rutinoside, kaempferol3-0-β rutinoside, rutin, kaempferol-3-L-rhamno-Dglucoside, azaridine, bakayanin, bakalactone, margosine, azadirone, azadiradione, epoxyazadiradione, ohchinol, ohchinin, ohchinolal, ohchinolides A and B, nimbolinin B, 1-desacetylnimboline B, nimbolidins A and B, triterpene B, meliacins A1,A2, B1, B2, sendanin, sendenal, 1-cinnamoylmelianolone, meliantriol, melianone, melianol, lupeol, β-sitosterol, catechin, vanillin, cinnamic acid, salannal, meliacarpinin E	analgesic, immunomodulatory, antifeedant, antifungal, antibacterial, antiviral, cytotoxic, anthelminthic, antilithic, antifertility	[184,185,186]
*Cissampelos pareira*	hayatine, hayatinine, hayatidine, quercitol, sterol, eepeerine, berberine, cissampeline, pelosine, cycleamine, menismin iodine, cissamin chloride, pareirin, cissamine chloride, cissampareine, dehydrodicentrine, dicentrine and insularine	antipyretic, anti-inflammatory, antiarthritic, antiulcer, antidiabetic, anticancer, antifertility, antimicrobial, antioxidant, antivenom, antimalarial, and immunomodulatory	[187,188]
*Ficus palmata*	germanicol acetate, vanillic acid, psoralenoside methyl ether, rutin	hepatoprotective, nephroprotective, antiulcer and anticoagulant	[189]
*Boerhavia diffusa*	eupalitin, eupalitin-3-O-β-Dgalactopyranos, eupatilin-7-O-β-Dgalactopyranoside, eupatilin 7-O-α-Lrhamnopyranosyl (1→2) α-Lrhamnopyranosyl (1→6)- β-Dgalactopyranoside, quercetin-3-O- β-Dglucopyranoside- 7-O- β-Dglucopyranoside, -3-O-α-Lrhamnopyranosyl (1→6)-β-Dgalactopyranoside, kaempferol, quercetin, borhavone, punarnavoside, alkamide, ferulic acid, gentisic acid, caffeoyltartaric acid, boerhaavic acid, boeravinone, coccineon, isomenthone, limonene, menthol, phellandrene, safranal, α-pinene, geranylacetone, eugenol, stigmasterol, campesterol, β-sitosterol	antidiabetic, immunosuppressive, nephroprotective, antilithiatic, antioxidant, hepatoprotective, antiviral, anticancer, cardiovascular	[190,191,192,193]
*Cedrus deodara*	wikstromal, matairesinol, dibenzylbutyrolactol, cedrin, taxifolin, cedeodarin, dihydromyricetin, cedrinoside, deodardione, diosphenol, limonenecarboxylic acid, deodarin, sitosterol, deodarone, atlantone, α-himacholone, β-himacholone, α-pinene, β-pinene, myrcene, himachalene, cis-atlantone, α-atlantone	anti-inflammatory, analgesic, anti-spasmodic, immunomodulatory, anti-hyperglycemic, anticancer, antibacterial	[194]
*Plantago lanceolata*	pyrocatechol, picatechin, vanillin, verbascoside, taxifolin, luteolin 7-glucoside, hesperidin, hyperoside, apigenin 7-glucoside, pinoresinol, eriodictyol, quercetin, luteolin, kaempferol, apigenin, baicalein, plantaginin, aucubin, indicain, plantagonin	antiulcerative, antidiabetic, antidiarrhoeal, anti-inflammatory, anticancer, antinociceptive, antioxidant, anti-fatigue, antibacterial, antiviral	[195,196]
*Picrorhiza kurroa*	veronicoside, pikuroside, cucurbitacins, 4-hydroxy-3-methoxy acetophenone, apocyanin, drosin	hepatoprotective, anticholestatic, antioxidant, anti-inflammatory, anticancer, antiviral, hepatoprotective, anticonvulsant, nephroprotective	[197]
*Rumex dentatus*	rumejaposides E, cassialoin, emodin	astringent, antioxidant, antibacterial	[198]
*Persicaria hydropiper*	catechin, epicatechin, hyperin, isoquercitrin, isorhamnetin, kaempferol, quercetin, quercitrin, rhamnazin, rutin, 3-*β*-angeloyloxy-7-epifutronolide, 7-ketoisodrimenin, changweikangic acid A, dendocarbin L, fuegin, futronolide, polygonumate, winterin, confertifolin, isodrimeninol	antioxidant, antibacterial, antifungal, anthelminthic, cytotoxic, anti-inflammatory, antifertility, neuroprotective	[199]
*Aconitum heterophyllum*	aconitine, heterophylline A, heterophylline B, heteratisine, heterophyllisine, heterophyllidine, mesaconitine, 3 acetylaconitine, atidine, isoatisine, hetidine, hetsinone, benzoylheteratisine, aconitic acid, N-Diethyl-N-formyllyaconitine, O-methylaconitine, methyl-N-succinoylanthranilate, hypaconitine, benzoylaconine, benzoylmesaconine, benzoylhypaconine, 6-dehydroacetylsepaconitine, 13-hydroxylappaconitine	antidiarrheal, expectorant, diuretic, hepatoprotective, antipyretic, analgesic, antioxidant, alexipharmic, anodyne, anti-atrabilious, immunostimulant, febrifuge, anthelmintic, anti-cancerous, anti-emetic, anti-inflammatory, anti-flatulent, anti-periodic, anti-phlegmatic, anti-diabetic, antifungal, antimicrobial, antiviral and carminative	[200,201,202,203]
*Prunus persica*	lutein, β-cryptoxanthin, β-carotene, champagne, hexahydroxydiphenic acid, gallic acid, protocatechuic acid, guercetin, kaempferol, isorhamnetin, myricetin, cyanidin-3-O-glucoside, cyanidin-3-O-rutinoside, catechin, epicatechin	antioxidant, antimicrobial, antidiabetic, anti-inflammatory, cardioprotective, neuroprotective, anticancer	[204]
*Galium aparine*	gallic acid, caffeic acid, isoquercitrin, rutin, quercetin, luteolin, asperulosidic acid, deacetylasperulosidic acid, β-sitosterol, daucosterol, vanillin, γ-sitosterol	antioxidant, antimicrobial, anticancer, hepatoprotective, antifeedant	[205,206,207]
*Salix alba*	anthocyanins, p-hydroxybenzoic, gallic acid, Gentisic acid, sisymbrifolin	antibacterial, antifungal, antioxidant	[208]
*Aesculus indica*	quercetin, mandelic acid	Immunomodulatory	[209,210]
*Dodonaea viscosa*	aliarin, pinocembrin, viscosol, sakuranetin, isokaempferide, dodoviscins A-J, isorhamnetin, quercetin, jegosapogenol, β-pinene, myrcene, limonene, p-cymene, citronellal, linalool, linalyl acetate, Υ-terpineol, geraniol, α-spinasterol, 4-hydroxy-3,5-diprenylbenzaldehyde, β-sitosterol, stearic acid, syringic acid, fraxetin, cleomiscosin A, cleomiscosin C, and β-sitosterol β -D-glucoside	Antidiabetic, antimicrobial, antioxidant, cytotoxic, antifertility, anti-inflammatory, analgesic, antiulcer, anti-spasmodic	[211]
*Bergenia ciliata*	bergenin, tannic acid, gallic acid, catechin, β-sitosterol, arbutin, afzelechin, quercetin 3-o-β-D xylopyranoside, quercetin 3-o-α-L-arbinofuranoxide, Linalool,	antidiabetic, anticancer, antiulcer, antibacterial, antimalarial, antifungal, antiviral	[212]
*Verbascum thapsus*	verbascoside, aucubin, harpagoside, ajugol, isocatalpol, methylcatalpol, 6-O-α-L-rhamnopyranosylcatalpol, saccatoside, harpagide, ningpogenin, 10-deoxyeucommiol, jioglutolideforsythoside B, alyssonoside, arenarioside, saikogenin A, thapsuine A, β-spinasterol, veratric acid	antioxidant, antimicrobial, antiviral, anticancer, antihyperlipidemic	[213]
*Datura stramonium*	atropine, scopoline, 3-(hydroxyacetoxy) tropan, 3-hydroxy-6-(2-methylbutyryloxy) tropan, aponorscopolamine, 7-hydroxyhyoscyamin, aponorscopolamine, aposcopolamine, hygrine, hyoscyamine, littorine, meteloidine, scopine scopolamine, tropinone, tropine	antimicrobial, analgesic, anti-asthmatic, anticancer, antioxidant	[214]
*Urtica dioica*	acetylcholine, histamine, and 5-hydroxytryptamine, formic acid, histamine, serotonin, carvacrol, carvone, isolectins	anti-inflammatory, analgesic, antiviral, hepatoprotective, anti-colitis,	[215]
*Sambucus wightiana*	-	Antimicrobial	[216]
*Viburnum grandiflorum*	luteolin 3′-O-b-d-xylopyranosyl(1!2)-O-b-d-glucopyranoside, quercetine	Antifungal	[217,218]

**Table 7:** Biological activities and mechanism of action of some of the phytochemicals.

Phytochemical	PubChem CID	Chemical Formula	Biological Activities	Mechanism of Action	Reference(s)
Rutin	5280805	C_27_H_30_O_16_	Modifies the cognitive and various behavioral symptoms of neurodegenerative diseases	Effects processing, aggregation and action of amyloid beta (Aβ); shift of the oxidant–antioxidant balance allied with neuronal cell loss	[221]
			Antihyperglycemic	Decrease in carbohydrates’ absorption from the small intestine, inhibition of tissue gluconeogenesis, an increase of tissue glucose uptake, stimulation of insulin secretion from beta cells, and protecting Langerhans’ islet against degeneration	[222]
Quercetin	5280343	C_15_H_10_O_7_	Anti-inflammatory	Inhibits LPS-induced mRNA levels of cytokines in colloid cells, such as tumor necrosis factor (TNF)-a and IL-1a	[223]
			Anticancer	Suppression of many kinases involved in the growth of cancer cells, proliferation and metastasis	[224]
Lupeol	259846	C_30_H_50_O	Anti-inflammatory	Suppresses and alters the phagocytic activity of macrophages and T lymphocytes, and suppresses CD4+ T cell mediated cytokine generation	[225]
Apigenin	5280443	C_15_H_10_O_5_	Anxiolytic	Inhibits the binding of flunitrazepam to brain membranes without influencing the binding of muscimol to GABA_A_ receptors.	[226]
			Anti-inflammatory	Downregulates the expression of IL-1β and TNF-α in LPS-stimulated mouse macrophages and human monocytes	[227]
Calotropin	16142	C_29_H_40_O_9_	Cytotoxic	Upregulated the expression of p27 leading to cell arrest by downregulating the G2/M regulatory proteins, cyclins A and B, and by upregulating the cdk inhibitor, p27	[228]
α-hederin	319412227	C_41_H_66_O_12_	Anticancer	Induces depolarization of mitochondrial membrane potential which released Apaf-1 and cytochrome c from the intermembrane space into the cytosol, where they promoted caspase-3 and caspase-9 activation	[229]
Luteolin	5280445	C_15_H_10_O_6_	Antibacterial	Disrupts the integrity of the bacterial cell membrane and cell wall, resulting in the leakage of cell contents and damage to the barrier function of the cell wall and membrane	[230]
Rupesin	134715087	C_15_H_22_O_5_	Antitumor	Inhibits the proliferation of Glioma Stem Cells (GSCs)	[231]
Kaempferol	5280863	C_15_H_10_O_6_	Anticancer	Induces apoptosis, cell cycle arrest at the G2/M phase, downregulation of Epithelial–Mesenchymal Transition (EMT)-related markers, and phosphoinositide 3-kinase/protein kinase B signaling pathways	[232]
Rottlerin	5281847	C_30_H_28_O_8_	Antitumor	Sensitizes MCF-7 breast cancer cells to TRIAL-mediated apoptosis by PKC*δ*-dependent inhibition of the transcription factor nuclear factor *κ*B (NF*κ*B),	[233]
Heterophylline	251575	C_22_H_26_N_2_O_4_	Alzheimer’s disease	Inhibits muscle-contracting enzymes acetylcholineatrase and butyrlcholinestrase	[234]

## Data Availability

Not applicable.

## References

[1] Balaji S.N., Chakravarthi V.P. (2010). Ethnoveterinary practices in India: A review. Vet. World.

[2] Wani Z.A., Pant S. (2020). Ethnomedicinal study of plants used to cure skin diseases and healing of wounds in Gulmarg Wildlife Sanctuary (GWLS), Jammu and Kashmir. Indian J. Tradit. Knowl..

[3] Mukherjee P.K., Harwansh R.K., Bahadur S., Banerjee S., Kar A. (2017). Evidence-based validation of Indian traditional medicine: Way forward. From Ayurveda to Chinese Medicine.

[4] Nema N.K., Dalai M.K., Mukherjee P.K. (2011). Ayush herbs and status que in herbal industries. Pharma Rev..

[5] Mukherjee P.K., Venkatesh P., Ponnusankar S. (2010). Ethnopharmacology and integrative medicine—Let the history tell the future. J. Ayurveda Integr. Med..

[6] Sharma R., Patki P. (2010). Double-blind, placebo-controlled clinical evaluation of an Ayurvedic formulation (GlucoCare capsules) in non-insulin dependent diabetes mellitus. J. Ayurveda Integr..

[7] Mukherjee P.K. (2002). Quality Control of Herbal Drugs—An Approach to Evaluation of Botanicals.

[8] Negi V.S., Maikhuri R.K., Chandra A., Maletha A., Dhyani P.P. (2018). Assessing sustainability of farming systems in mountain agroecosystems of Western Himalaya, India. Agroecol. Sustain. Food Syst..

[9] Negi V.S., Maikhuri R.K., Rawat L.S., Vashishtha D.P. (2010). The livestock production system in a village ecosystem in the Rawain valley, Uttarakhand, Central Himalaya. Int. J. Sustain. Dev. World Ecol..

[10] Rajkumari R., Nirmala R.K., Singh P.K., Das A.K., Dutta B.K., Pinokiyo A. (2014). Ethnoveterinary plants used by the Chiru tribes of Manipur, Northeast India. Indian J. Tradit. Knowl..

[11] Negi V.S., Maikhuri R.K., Rawat L.S. (2012). Paradigm and ecological implication of changing agricultural land-use: A case study from Govind Wildlife Sanctuary, Central Himalaya, India. J. Mt. Sci..

[12] Parthiban R., Vijayakumar S., Prabhu S., Yabesh J.G.E.M. (2015). Quantitative traditional knowledge of medicinal plants used to treat livestock diseases from Kudavasal taluk of Thiruvarur District, Tamil Nadu, India. Braz. J. Pharm..

[13] Xiong Y., Long C. (2020). An ethnoveterinary study on medicinal plants used by the Buyi people in Southwest Guizhou, China. J. Ethnobiol. Ethnomed..

[14] Eshetu G.R., Dejene T.A., Telila L.B., Bekele D.F. (2015). Ethnoveterinary medicinal plants: Preparation and application methods by traditional healers in selected districts of southern Ethiopia. Vet. World.

[15] Phondani P.C., Maikhuri R.K., Kala C.P. (2010). Ethnoveterinary uses of medicinal plants among traditional herbal healers in Alaknanda catchment of Uttarakhand, India. Afr. J. Trad. Compl. Alter. Med..

[16] Abebe D., Ayehu A. (1993). Medicinal Plants and Health Practices of Northern Ethiopia.

[17] Mathias E., McCorkle C.M., Bunders J., Haverkort B., Hiemstra W. (1997). Animal health. Biotechnology: Building on Farmers’ Knowledge.

[18] Raut A., Tillu G., Vaidya A.D.B. (2016). Reverse pharmacology effectuated by studies of Ayurvedic products for arthritis. Curr. Sci..

[19] Patwardhan B., Mashelkar R.A. (2009). Traditional medicine-inspired approaches to drug discovery: Can Ayurveda show the way forward?. Drug Discov. Today.

[20] Surh Y.J. (2013). Reverse pharmacology applicable for botanical drug development—Inspiration from the legacy of traditional medicine. J. Trad. Compl. Med..

[21] Kunwar R.M., Nepal B.K., Kshhetri H.B., Rai S.K., Bussmann R.W. (2006). Ethnomedicine in Himalaya: A case study from Dolpa, Humla, Jumla and Mustang districts of Nepal. J. Ethnobiol. Ethnomed..

[22] Sharma P.K., Singh V. (1989). Ethnobotanical studies in Northwest and Trans-Himalaya: Ethnoveterinary medicines used in Jammu and Kashmir, India. J. Ethnophar..

[23] Bhat M.N., Singh B., Surmal O., Singh B., Shivgotra V., Musarella C.M. (2021). Ethnobotany of the Himalayas: Safeguarding medical practices and traditional uses of Kashmir regions. Biology.

[24] Singh B., Singh S., Kishor A., Singh B. (2021). Traditional usage of medicinal plants in humans and animal health care and their chemical constituents from hills and valleys of Jammu province, Western Himalaya. Indian J. Nat. Prod. Res..

[25] Abass Z., Ahmad J., Ahmad I. (2015). Socio-economic and educational status of tribal (Gujjar and Bakerwal) of Jammu and Kashmir: An Overview. Int. J. Hum. Soc. Stud..

[26] Wani Z.A., Pant S., Singh B. (2021). Descriptive study of plant resources in context of ethnomedicinal relevance of indigenous flora; a case study from Rajouri-Poonch region of Himalaya. Ethnobot. Res. Appl..

[27] Negi V.S., Pathak R., Thakur S., Joshi R.K., Bhatt I.D., Rawal R.S. (2021). Scoping the Need of Mainstreaming Indigenous Knowledge for Sustainable Use of Bioresources in the Indian Himalayan Region. Environ. Manag..

[28] Bhatia H., Sharma Y.P., Manhas R.K., Kumar K. (2014). Ethnomedicinal plants used by the villagers of district Udhampur, J&K, India. J. Ethnophar..

[29] Bisht N.S., Khajuria A.K. (2014). Ethno-medicinal plants of Tehsil, Kathua, Jammu & Kashmir. J. Mount. Res..

[30] Gairola S., Sharma J., Bedi Y.S. (2014). A cross-cultural analysis of Jammu, Kashmir and Ladakh (India) medicinal plant use. J. Ethnopharmacol..

[31] Rao P.K., Hasan S.S., Bhellum B.L., Manhas R.K. (2015). Ethnomedicinal plants of Kathua district, J&K, India. J. Ethnopharmacol..

[32] Kumar K., Sharma Y.P., Manhas R.K., Bhatia H. (2015). Ethnomedicinal plants of Shankaracharya Hill, Srinagar, J&K, India. J. Ethnopharmacol..

[33] Shah A., Bharati K.A., Ahmad J., Sharma M.P. (2015). New ethnomedicinal claims from Gujjar and Bakerwals tribes of Rajouri and Poonch districts of Jammu and Kashmir, India. J. Ethnopharmacol..

[34] Tali B.A., Khuroo A.A., Ganie A.H., Nawchoo I.A. (2019). Diversity, distribution and traditional uses of medicinal plants in Jammu and Kashmir (J&K) state of Indian Himalayas. J. Herb. Med..

[35] Singh B., Singh B., Kishor A., Singh S., Bhat M.N., Surmal O., Musarella C.M. (2020). Exploring plant-based ethnomedicine and quantitative ethnopharmacology in protected area: Ethnobotanical study of medicinal plants utilized by population of Jasrota Hill in Western Himalaya, India. Sustainability.

[36] Jan M., Mir T.A., Ganie A.H., Khare R.K. (2021). Ethnomedicinal use of some plant species by Gujjar and Bakerwal community in Gulmarg Mountainous Region of Kashmir Himalaya. Ethnobot. Res. Appl..

[37] Heinrich M., Edwards S., Moerman D.E., Leonti M. (2009). Ethnopharmacological field studies: A critical assessment of their conceptual basis and methods. J. Ethnopharmacol..

[38] Edwards S., Nebel S., Heinrich M. (2005). Questionnaire surveys: Methodological and epistemological problems for field-based ethnopharmacologists. J. Ethnopharmacol..

[39] Negi V.S., Pathak R., Sekar K.C., Rawal R.S., Bhatt I.D., Nandi S.K., Dhyani P.P. (2018). Traditional knowledge and biodiversity conservation: A case study from Byans Valley in Kailash Sacred Landscape, India. J. Environ. Plan. Manag..

[40] Ribeiroa R.V., Bieskia I.G.C., Baloguna S.O., Martins D.T.O. (2017). Ethnobotanical study of medicinal plants used by Ribeirinhos in the North Araguaia microregion, Mato Grosso. J. Ethnopharmacol..

[41] Jain S.K., Rao R.R. (1976). Handbook of Field and Herbarium Methods.

[42] Singh N.P., Singh D.K., Uniyal B.P. (2002). Flora of Jammu and Kashmir.

[43] Swami A., Gupta B.K. (1998). Flora of Udhampur.

[44] Sharma B.M., Kachroo P. (1982). Flora of Jammu and Plants of Neighbourhood Volumes I–II.

[45] Phillips O., Gentry A.H. (1993). The useful plants of Tambopata, Peru, II: Additional hypothesis testing in quantitative ethnobotany. Econ. Bot..

[46] Zenderland J., Hart R., Bussmann R., Zambrana M.P., Sikharulidze S., Kikodze D., Tchelidze D., Khutsishvili M., Batsatsashvili K. (2019). The Use of “Use Value”: Quantifying Importance in Ethnobotany. Econ. Bot..

[47] Phillips O., Gentry A.H., Reynel C., Wilki P., Gavez–Durand C.B. (1994). Quantitative ethnobotany and Amazonian conservation. Conserv. Biol..

[48] Musa M.S., Abdelrasool F.E., Elsheikh E.A., Ahmed L.A.M.N., Mahmoud A.L.E., Yagi S.M. (2011). Ethnobotanical study of medicinal plants in the Blue Nile State, South-eastern Sudan. J. Med. Plants Res..

[49] Heinrich M., Ankli A., Frei B., Weimann C., Sticher O. (1998). Medicinal plants in Mexico: Healers’ consensus and cultural importance. Soc. Sci. Med..

[50] Amjad M.S., Qaeem M.F., Ahmad I., Khan S., Chaudhari S.K., Malik N.Z., Shaheen H., Khan A.M. (2017). Descriptive study of plant resources in the context of the ethnomedicinal relevance of indigenous flora: A case study from Toli Peer National Park, Azad Jammu and Kashmir, Pakistan. PLoS ONE.

[51] Majeed M., Bhatti K.H., Amjad M.S., Abbasi A.M., Bussmann R.W., Nawaz F., Rashid A., Mahmood A., Khan W.M., Ahmad K.S. (2020). Ethno-veterinary uses of Poaceae in Punjab, Pakistan. PLoS ONE.

[52] Munir M., Sadia S., Khan A., Rahim B.Z., Nayyar B.G., Ahmad K.S., Khan A.M., Fatima I., Qureshi R. (2022). Ethnobotanical study of Mandi Ahmad Abad, District Okara, Pakistan. PLoS ONE.

[53] Dar G.H., Malik A.H., Khuroo A.A. (2014). A Contribution to the Flora of Rajouri and Poonch Districts in the Pir Panjal Himalaya (Jammu & Kashmir), India. Check List.

[54] Yineger H., Kelbessa E., Bekele T., Lulekal E. (2007). Ethnoveterinary medicinal plants at Bale Mountains National Park, Ethiopia. J. Ethnopharmacol..

[55] Khattak N.S., Nouroz F., Rahman I., Noreen S. (2015). Ethno veterinary uses of medicinal plants of district Karak, Pakistan. J. Ethnopharmacol..

[56] Sharafatmandrad M., Mashizi A.K. (2020). Ethnopharmacological study of native medicinal plants and the impact of pastoralism on their loss in arid to semiarid ecosystems of southeastern Iran. Sci. Rep..

[57] Okach D.O., Nyunja A.R.O., Opande G. (2013). Phytochemical screening of some wild plants from Lamiaceae and their role in traditional medicine in Uriri District—Kenya. Int. J. Herb. Med..

[58] Rodriguez-Chavez J.L., Egas V., Linares E., Bye R., Hernandez T., Espinosa-Garcia F.J., Delgado G. (2017). Mexican arnica (*Heterotheca inuloides* Cass. Asteraceae: Asteraceae): Ethnomedical uses, chemical constituents and biological properties. J. Ethnopharmacol..

[59] Tewari D., Mocan A., Parvanov E.D., Sah A.N., Nabavi S.M., Huminiecki L., Ma Z.F., Lee Y.Y., Horbanczuk J.O., Atanasov A.G. (2017). Ethnopharmacological approaches for therapy of jaundice: Part II. Highly used plant species from Acanthaceae, Euphorbiaceae, Asteraceae, Combretaceae, and Fabaceae families. Front. Pharmacol..

[60] Saleh E.I.M.M., Van Staden J. (2018). Ethnobotany, phytochemistry and pharmacology of *Arctotis arctotoides* (L.f.) O. Hoffm.: A review. J. Ethnopharmacol..

[61] Gao T., Yao H., Song J., Zhu Y., Liu C., Chen S. (2010). Evaluating the feasibility of using candidate DNA barcodes in discriminating species of the large Asteraceae family. BMC Evol. Biol..

[62] Pant S., Samant S.S. (2010). Ethnobotanical observations in Mornaula Research Forest of Kumoun, West Himalaya, India. Ethnobot. Leafl..

[63] Chekole G., Asfaw Z., Kelbessa E. (2015). Ethnobotanical study of medicinal plants in the environs of Tara-gedam and Amba remnant forests of Libo Kemkem District, northwest Ethiopia. J. Ethnobiol. Ethnomed..

[64] Cheikhyoussef A., Summers R.W., Kahaka G. (2015). Qualitative and quantitative analysis of phytochemical compounds in Namibian *Myrothamnus flabellifolius*. Int. Sci. Technol. J. Namib..

[65] Malik K., Ahmad M., Zafar M., Ullah R., Mehmood H.M., Parveen B., Rashid N., Sultana S., Shah S.N., Lubna (2019). An ethnomedicinal study of medicinal plants used to treat skin diseases in northern Pakistan. BMC Complement. Altern. Med..

[66] Bano A., Ahmad M., Hadda T.B., Saboor A., Sultana S., Zafar M., Khan M.P.K., Arshad M., Ashraf M.A. (2014). Quantitative ethnomedicinal study of plants used in the skardu valley at high altitude of Karakoram-Himalayan range, Pakistan. J. Ethnobiol. Ethnomedicine.

[67] Yaseen G., Ahmad M., Sultana S., Alharrasi A.S., Hussain J., Zafar M., Rehman S. (2015). Ethnobotany of Medicinal Plants in the Thar Desert (Sindh) of Pakistan. J. Ethnopharmacol..

[68] Mahishi P., Srinivasa B.H., Shivanna M.B. (2005). Medicinal plant wealth of local communities in some villages in Shimoga District of Karnataka, India. J. Ethnopharmacol..

[69] Bose D., Roy J.G., Mahapatra S.D., Datta T., Mahapatra S.D., Biswas H. (2015). Medicinal plants used by tribals in Jalpaiguri district, West Bengal, India. J. Med. Stud..

[70] Faruque M., Uddin S., Barlow J., Hu S., Dong S.Q., Li X., Hu X. (2018). Quantitative ethnobotany of medicinal plants used by indigenous communities in the Bandarban district of Bangladesh. Front. Pharmacol..

[71] Raj A.J., Biswakarma S., Pala N.A., Shukla G., Vineeta, Kumar M., Chakravarty S., Bussmann R.M. (2018). Indigenous uses of ethnomedicinal plants among forest-dependent communities of Northern Bengal, India. J. Ethnobiol. Ethnomed..

[72] Pala N.A., Sarkar B.C., Shukla G., Chettri N., Deb S., Bhat J.A., Chakravarty S. (2019). Floristic composition and utilization of ethnomedicinal plant species in home gardens of the Eastern Himalaya. J. Ethnobiol. Ethnomed..

[73] Giday M., Asfaw Z., Woldu Z. (2009). Medicinal plants of the Meinit ethnic group of Ethiopia: An ethnobotanical study. J. Ethnopharmacol..

[74] Saxena M., Saxena S., Nema R., Singh D., Gupta A. (2013). Phytochemistry of medicinal plants. J. Pharmacogn. Phytochem..

[75] Ayyanar M., Ignacimuthu S. (2011). Ethnobotanical survey of medicinal plants commonly used by Kani tribals in Tirunelveli hills of Western Ghats, India. J. Ethnopharmacol..

[76] Yabesh J.E.M., Prabhu S., Vijayakumar S. (2014). An ethnobotanical study of medicinal plants used by traditional healers in silent valley of Kerala, India. J. Ethnopharmacol..

[77] Rahman I.U., Ijaz F., Iqbal Z., Afzal A., Ali N., Afzal M., Khan M.A., Muhammad S., Qadir G., Asif M.A. (2016). Novel Survey of the Ethno Medicinal Knowledge of Dental Problems in Manoor Valley (Northern Himalaya), Pakistan. J. Ethnopharmacol..

[78] Mukherjee P.K., Wahile A. (2006). Integrated approaches towards drug development from Ayurveda and other Indian system of medicines. J. Ethnopharmacol..

[79] Majid A., Ahmad H., Saqib Z., Rahman I., Khan U., Alam J., Shah A.H., Jan S.A., Ali N. (2019). Exploring threatened traditional knowledge; ethnomedicinal studies of rare endemic flora from Lesser Himalayan region of Pakistan. Braz. J. Phar..

[80] Appiah K.S., Mardani H.K., Osivand A., Kpabitey S., Amoatey C.A., Oikawa Y., Fujii Y. (2017). Exploring alternative use of medicinal plants for sustainable weed management. Sustainability.

[81] Linh D.T.T., Duong H.T., Hiep N.T., Huyen P.T., Khoi N.M., Long D.D. (2020). Simultaneous quantification of Hederacoside C and α-hederin in *Hedera nepalensis* K. Koch using HPLC-UV. VNU J. Sci. Med. Pharm. Sci..

[82] Madikizela B., Ndhlala A.R., Finnie J.F., Van Staden J. (2012). Ethnopharmacological study of plants from Pondoland used against diarrhoea. J. Ethnopharmacol..

[83] Leonti M., Sticher O., Heinrich M. (2003). Antiquity of medicinal plant usage in two Macro-Mayan ethnic groups (Mexico). J. Ethnopharmacol..

[84] Rahman S., Ismail M., Shah M.R., Iriti M., Muhammad S. (2015). GC/MS analysis, free radical scavenging, anticancer β glucuronidase inhibitory activities of *Trillium govanianum* rhizome. Bangladesh J. Pharm..

[85] Leonti M. (2011). The future is written: Impact of scripts on the cognition, selection, knowledge and transmission of medicinal plant use and its implications for ethnobotany and ethnopharmacology. J. Ethnopharmacol..

[86] Ch M.I., Khan M.A., Hanif W. (2006). Ethnoveterinary Medicinal Uses of Plants of from Samahni Valley District Bhimber, (Azad Kashmir) Pakistan. Asian J. Plant Sci..

[87] Sharma R., Manhas R.K., Magotra R. (2012). Ethnoveterinary remedies of diseases among milk yielding animals in Kathua, Jammu and Kashmir, India. J. Ethnopharmacol..

[88] Khuroo A.A., Malik A.H., Dar A.R., Dar G.H., Khan Z.S. (2007). Ethno-veterinary medicinal uses of some plant species by the Gujjar tribe of the Kashmir Himalaya. Asian J. Plant Sci..

[89] Khan M.A., Khan M.A., Mujtaba G., Hussain M. (2012). Ethnobotanical study about medicinal plants of Poonch valley Azad Kashmir. J. Anim. Plant Sci..

[90] Harsha V.H., Shripathi V., Hedge G.R. (2005). Ethnoveterinary practices in Uttara Kannada district of Karnataka. Indian J. Tradit. Knowl..

[91] Yadav S.S., Bhukal R.K., Bhandoria M.S., Ganie S.A., Gulia S.K., Raghav T.B.S. (2014). Ethnoveterinary Medicinal plants of Tosham block of district Bhiwani (Haryana) India. J. Appl. Pharm. Sci..

[92] Nigam G., Sharma N.K. (2010). Ethnoveterinary plants of Jhansi district, Uttar Pradesh. Indian J. Tradit. Knowl..

[93] Aziem S., Chamola B.P., Mahato S., Pala N.A. (2013). Utilization and traditional knowledge of ethnoveterinary medicinal plants in Tehri district of Garhwal Himalaya, India. Int. J. Indig. Med. Plants.

[94] Narayana V.L., Narasimharao G.M. (2015). Plants used in Ethnoveterinary Medicine by Tribals of Visakhapatnam and Vizianagarm Districts, Andhra Pradesh, India. Int. J. Pure Appl. Biosci..

[95] Chen G., Yang M., Song Y., Lu Z., Zhang J., Huang H., Guan S., Wu L., Guo D. (2008). Comparative analysis on microbial and rat metabolism of ginsenoside Rb1 by high-performance liquid chromatography coupled with tandem mass spectrometry. Biomed. Chromatogr..

[96] Ahmad M., Sultana S., Fazl-i-Hadi S., Ben Hadda T., Rashid S., Zafar M., Khan M.A., Khan M.P.Z., Yaseen G. (2014). An Ethnobotanical study of Medicinal Plants in high mountainous region of Chail valley (District Swat-Pakistan). J. Ethnobiol. Ethnomedicine.

[97] Kumar A.B.S., Lakshman K., Jayaveera K.N., Nandeesh R., Manitripathi S.N., Krishna V., Manjunath M., Suresh M.V. (2009). Estimation of Rutin and Quercitin in *Amaranthus viridis* L. by High Performance Layer Chromatography (HPLC). Ethnobot. Leafl..

[98] Iqbal M.J., Hanif S., Mahmood Z., Anwar F., Jamil A. (2012). Antioxidant and antimicrobial activities of Chowlai (*Amaranthus viridis* L.) leaf and seed extracts. J. Med. Plants Res..

[99] Carminate B., Martin G.B., Barcelos R.M., Gontijo I. (2012). Evaluation of antifungal activity of *Amaranthus viridis* L. (Amaranthaceae) on *Fusariosis* by *Piper nigrum* L. and on Anthracnose by *Musa* sp. Agric. J..

[100] Kumar A.B.S., Lakshman K., Jayaveera K.N., Nandeesh R., Manoj B., Ranganayakula D. (2010). Comparative in vitro anthelminthic activity of three plants from the Amaranthaceae family. Arch. Biol. Sci..

[101] Ahmad M., Mohiuddin O.A., Mehjabeen N.J., Anwar M., Habib S., Alam S.M., Baig I.A. (2012). Valuation of spasmolytic and analgesic activity of ethanolic extract of *Chenopodium album* (Linn.) and its fraction. Med. Plants Res..

[102] Akhtar M.B., Iqbal Z., Khan M.N. (1999). Evaluation of anthelmintic activity of *Chenopodiumalbum* (Bathu) against nematodes in sheep. Int. J. Agric. Biol..

[103] Bylka W., Kowalewski Z. (1997). Flavonoids in *Chenopodium album* L. and *Chenopodium opulifolium* L. Herba Pol..

[104] Goyal B.R., Goyal R.K., Mehta A.A. (2007). Phyto-pharmacology of *Achyranthes aspera*: A review. Pharmacogn. Rev..

[105] Prasad S., Bhattacharya I.C. (1961). Pharmacognostical studies of *Achyranthes aspera* Linn. J. Sci. Ind. Res..

[106] Kapoor V.K., Singh H. (1966). Isolation of betain from *Achyranthes aspera* Linn. Indian J. Chem..

[107] Fossen T., Pedersen A.T., Andersen O.M. (1998). Flavonoids from red onion (*Allium cepa*). Phytochemistry.

[108] Nasri S., Anoush M., Khatami N. (2012). Evaluation of analgesic and anti-inflammatory effects of fresh onion juice in experimental animals. Afr. J. Pharm. Pharmacol..

[109] Sakakibara H., Yoshino S., Kawai Y., Terao J. (2008). Antidepressant-like effect of onion (*Allium cepa* L.) powder in a rat behavioral model of depression. Biosci. Biotechnol. Biochem..

[110] El-Saber B.G., Magdy B.A., Wasef G.L., Elewa Y.H.A., Al-Sagan A.A., Abd El-Hack M.E., Taha A.E., Abd-Elhakim M.Y., Prasad D.H. (2020). Chemical Constituents and Pharmacological Activities of Garlic (*Allium sativum* L.): A Review. Nutrients.

[111] Thomson M., Ali M. (2003). Garlic (*Allium sativum*): A review of its potential use as an anti-cancer agent. Curr. Cancer Drug Targ..

[112] Tesfaye A., Mengesha W. (2015). Traditional uses, phytochemistry and pharmacological properties of garlic (*Allium sativum*) and its biological active compounds. Int. J. Sci. Res. Eng. Technol..

[113] Butola J.S., Vashistha R.K. (2013). An overview on conservation and utilization of *Angelica glauca* Edgew. in three Himalayan states of India. Med. Plants.

[114] Chopra R.N., Ayer S.L., Chopra I.C. (1992). Glossary of Indian Medicinal Plants.

[115] (1985). Wealth of India, Revised Volume 1.

[116] Hoult J.R.S., Paya M. (1996). Pharmacological and Biochemical activities of simple coumarins: Natural products with therauptical potential. Gen. Pharmacol. Vasc. Syst..

[117] Badgujar S.B., Patel V.V., Bandivdekar A.H. (2014). *Foeniculum vulgare* Mill: A review of its botany, phytochemistry, pharmacology, contemporary application, and toxicology. BioMed Res. Int..

[118] Parejo I., Jauregui O., Sanchez-Rabaneda F., Viladomat F., Bastida J., Codina C. (2004). Separation and characterization of phenolic compounds in fennel (*Foeniculum vulgare*) using liquid chromatography-negative electrospray ionization tandem mass spectrometry. J. Agric. Food Chem..

[119] Sharma K., Kharb R., Kaur R. (2011). Pharmacognostical aspects of *Calotropis procera* (Ait.) R.Br. Int. J. Pharm. Biol. Sci..

[120] Verma R., Satsangi G.P., Shrivastava J.N. (2010). Ethno-medicinal profile of different plant parts of *Calotropis procera* (Ait.) R.Br. Ethnobot. Leafl..

[121] Gupta S., Gupta B., Kapoor K., Sharma P. (2012). Ethnopharmacological potential of *Calotropis procera*: An overview. Int. Res. J. Pharm..

[122] Quazi S., Mathur K., Arora S. (2013). *Calotropis procera*: An overview of its phytochemistry and pharmacology. Indian J. Drugs.

[123] Jafri L., Saleem S., Kondrytuk T.P., Haq I.U., Ullah N., Pezzuto J.M., Mirza B. (2016). *Hedera nepalensis* K. Koch: A Novel Source of Natural Cancer Chemopreventive and Anticancerous Compounds. Phytother. Res..

[124] Hashmi W.J., Ismail H., Mehmood F., Mirza B. (2018). Neuroprotective, antidiabetic and antioxidant effect of *Hedera nepalensis* and lupeol against STZ + AlCl_3_ induced rats model. DARU J. Pharm. Sci..

[125] Al-Malki A.L., Abo-Golayel M.K., Abo-Elnaga G., Al-beshri H. (2013). Hepatoprotective effects of dandelion (*Taraxacum officinale*) against induced chronic liver cirrhosis. J. Med. Plants Res..

[126] Clare B.A., Conroy R.S., Spelman K. (2009). The diuretic effect in human subjects of an extract of *Taraxacum officinale* folium over a single day. J. Altern. Complement. Med..

[127] Mir M.A., Sawhney S.S., Jassal M.M.S. (2013). Qualitative and quantitative analysis of phytochemicals of *Taraxacum officinale*. Wudpecker J. Pharm. Pharmocol..

[128] Grauso L., Emrick S., De Falco B., Lanzotti V., Bonamoni G. (2019). Common dandelion: A review of its botanical, phytochemical and pharmacological profiles. Phytochem. Rev..

[129] Jeon H.J., Kang H.J., Jung H.J., Kang Y.S., Lim C.J., Kim Y.M., Park E.H. (2008). Anti-inflammatory activity of *Taraxacum officinale*. J. Ethnopharmacol..

[130] Mukhtar N., Iqbal K., Anis I., Malik A. (2002). Sphingolipids from *Conyza canadensis*. Phytochemistry.

[131] Shah N.Z., Khan M.A., Muhammad N., Azeem S. (2012). Antimicrobial and phytotoxic study of *Conyza canadensis*. J. Med. Plants Res..

[132] Al-Snafi A.E. (2017). Pharmacological and therapeutic importance of *Erigeron canadensis* (Syn. *Conyza canadensis*). Indo Am. J. Pharm. Sci..

[133] Govindan S.V., Bhattacharaya S.C. (1977). Alantolides and cyclocostunolides from *Saussurea lappa*. Indian J. Chem..

[134] Kumar S., Ahuja N.M., Juawanda G.S., Chhabra B.R. (1995). New guaianolides from *Saussurea lappa* roots. Fitoterapia.

[135] Cho J.Y., Park J., Yoo E.S., Baik K.U., Jung J.H., Lee J., Park M.H. (1998). Inhibitory effect of sesquiterpene lactones from *Saussurea lappa* on tumor necrosis factor-alpha production in murine macrophage like cells. Planta Med..

[136] Talwar K.K., Singh I.P., Kalsi P.S. (1991). A serquiterpenoid with plant growth regulatory activity from *Saussurea lappa*. Phytochemistry.

[137] Pandey M.M., Rastogi S., Rawat A.K.S. (2007). *Saussurea costus*: Botanical, chemical and pharmacological review of an ayurvedic medicinal plant. J. Ethnopharmacol..

[138] Lalla J.K., Hamrapurkar P.D., Mukherjee S.A., Thorat U.R. Sensitivity of HPTLC v/s HPLC for the analysis of alkaloid-Saussurine. Proceedings of the 54th Indian Pharmaceutical Congress.

[139] Tandi J., Sutrisna I.N.E., Pratiwi M., Handayani T.W. (2020). Potential test neuropathy *Sonchus arvensis* L. leaves on male rats (*Rattus norvegicus*) Diabetes mellitus. Pharmacogn. J..

[140] Xia D.Z., Yu X.F., Zhu Z.Y., Zou Z.D. (2011). Antioxidant and antibacterial activity of six edible wild plants (*Sonchus* spp.) in China. Nat. Prod. Res..

[141] Alkreathy H.M., Khan R.A., Khan M.R., Sahreen S. (2014). CCl4 induced genotoxicity and DNA oxidative damages in rats: Hepatoprotective effect of *Sonchus arvensis*. BMC Complement. Altern. Med..

[142] Akram M. (2013). Minireview on *Achillea millefolium* Linn. J. Membr. Biol..

[143] Applequist W.L., Moerman D.E. (2011). Yarrow (*Achillea millefolium* L.): A neglected panacea? A review of ethnobotany, bioactivity and biomedical research. Econ. Bot..

[144] Chandler R.F., Hooper S.N., Harvey M.J. (1982). Ethnobotany and phytochemistry of yarrow, *Achillea millefolium*, Compositae. Econ. Bot..

[145] Parra S.A., Gaur K., Ranawat L.S., Rather M.I. (2018). An overview of various aspects of plant *Berberis lycium* Royale. Am. J. Pharmacol. Sci..

[146] Shabir A., Shahzad M., Arfat Y., Ali L., Aziz R.S., Murtaza G., Waqar S.A., Alamgeer (2012). *Berberis lycium* Royle: A review of its traditional uses, phytochemistry and pharmacology. Afr. J. Pharm. Pharmacol..

[147] Sajid M., Khan M.R., Shah N.A., Shah S.A., Ismail H., Younis T., Zahra Z. (2016). Phytochemical, antioxidant and hepatoprotective effects of *Alnus nitida* bark in carbon tetrachloride challenged Sprague Dawley rats. BMC Complement. Altern. Med..

[148] Sajid M., Yan C., Li D., Merugu B., Negi H., Khan M.R. (2019). Potent anticancer activity of *Alnus nitida* against lung cancer cells; in vitro and in vivo studies. Biomed. Pharmacother..

[149] Siddiqui I.N., Ahmad V.U., Zahoor A., Ahmed A., Khan S.S., Khan A., Hassan Z. (2010). Two new diaryl heptanoids from *Alnus nitida*. Nat. Prod. Commun..

[150] Dejanovic G.M., Asllanaj E., Gamba M., Raguindin P.F., Itodo O.A., Minder B., Bussler W., Metzger B., Muka T., Glisic M. (2021). Phytochemical characterization of turnip greens (*Brassica rapa* ssp. *rapa*): A systematic review. PLoS ONE.

[151] Jan S.A., Shinwari Z.K., Malik M., Ilyas M. (2018). Antioxidant and anticancer activities of *Brassica rapa*: A review. MOJ Biol. Med..

[152] Nandeesh R., Kumar A., Lakshman K., Swamy V.B.N., Khan S., Ganapathy S. (2010). Evaluation of wound healing activity of *Buxus wallichiana* Bail. Asian J. Pharmacodyn. Pharmacokinet..

[153] Ata A., Naz S., Choudhary M.I., Atta-ur-Rahman N., Sener B., Turkoz S. (2002). New triterpenoidal alkaloids from *Buxus sempervirens*. Z. Nat. C.

[154] Atta-ur-Rahman, Ata A., Naz S., Choudhary M.I., Sener B., Turkoz S. (1999). New Steroidal Alkaloids from the roots of *Buxus sempervirens*. J. Nat. Prod..

[155] Bonini S.A., Premoli M., Tambaro S., Kumar A., Maccarinelli G., Memo M., Mastinu A. (2018). *Cannabis sativa*: A comprehensive ethnopharmacological review of a medicinal plant with a long history. J. Ethnopharmacol..

[156] Jugran A.K., Rawat S., Bhatt I.D., Rawal R.S. (2018). *Valeriana jatamansi*: An herbaceous plant with multiple medicinal uses. Phytother. Res..

[157] Prajapati R.P., Kalariya M., Parmar S.K., Sheth N.R. (2010). Phytochemical and pharmacological review of *Lagenaria siceraria*. J. Ayurveda Integr. Med..

[158] Tanaka R., Nakata T., Yamaguchi C., Wada S., Yamada T., Tokuda H. (2008). Potential anti-tumor-promoting activity of 3-Hydroxy-D: A-friedooleanan-2-one from the stem bark of *Mallotus philippinensis*. Planta Med..

[159] Kumar A., Patil M., Kumar P., Bhatti R.C., Kaur R., Sharma N.K., Singh A.N. (2021). *Mallotus philippensis* (Lam.) Mull. Arg. A review on its pharmacology and phytochemistry. J. Herbmed Pharm..

[160] Marwat S.K., Rehman F., Khan E.A., Baloch M.S., Sadiq M., Ullah I., Javaria S., Shaheen S. (2017). Review-*Ricinus communis*-Ethnomedicinal uses and pharmacological activities. Pak. J. Pharm. Sci..

[161] Pope G.S., Elcoate P.V., Simpson S.A., Andrews D.G. (1953). Isolation of an oestrogenic isoflavone (biochanin A) from redclover. Chem. Ind..

[162] Sabudak T., Guler N. (2009). *Trifolium* L. A review on its phytochemical and pharmacological profile. Phytother. Res..

[163] Oleszek W., Stochmal A. (2002). Triterpene saponins and flavonoids in the seeds of *Trifolium* species. Phytochemistry.

[164] Toppo F.A., Anand R., Pathak A.K. (2009). Pharmacological actions and potential uses of *Trigonella foenum graecum* L. A review. Asian J. Pharm. Clin. Res..

[165] Arunabha M., Bhattacharjee C. (2019). *Trigonella foenum-graecum*: A review of its traditional uses, phytochemistry and pharmacology. Int. J. Adv. Sci. Res..

[166] Gupta A., Behl T., Panichayupakaranan P. (2019). A review of phytochemistry and pharmacology profile of *Juglans regia*. Obes. Med..

[167] Al-Snafi A.E. (2018). Chemical constituents, nutritional, pharmacological and therapeutic importance of *Juglans regia*—A review. IOSR J. Pharm..

[168] Suva M.A. (2014). A Brief Review on *Vitex negundo* Linn: Ethnobotany, Phytochemistry and Pharmacology. Planta Act..

[169] Rastogi T., Kubde M., Farooqui I.A., Khadabadi S.S. (2017). A review on ethnomedicinal uses and phyto-pharmacology of anti-inflammatory herb *Vitex negundo*. Int. J. Pharm. Sci. Res..

[170] Nirja R., Sharma M.L. (2016). Antidiabetic and antioxidant activity of ethanolic extract of *Ajuga parviflora* Benth. (Lamiaceae) vern. Neelkanthi, Neelbati. Int. J. Pharm. Sci. Rev. Res..

[171] Khan P.M., Malik A., Ahmad S., Nawab H.F. (1999). Withanolides from *Ajuga parviflora*. J. Nat. Prod..

[172] Rahman N., Ahmad M., Riaz M., Mehjabeen J.N., Ahmad R. (2013). Phytochemical, antimicrobial, insecticidal and brine shrimp lethality bioassay of the crude methanolic extract of *Ajuga parviflora* Benth. Pak. J. Pharm. Sci..

[173] Rastogi R.P., Mehrotra B.N. (1991). Compendium of Indian Medicinal Plants.

[174] Jamal A., Siddiqui A., Tajuddin A., Jafri M.A. (2006). A review on gastric ulcer remedies used in Unani System of medicine. Nat. Prod. Rad..

[175] Akram M., Uzair M., Malik N.S., Mahmood A., Sarwer N., Madni A., Asif H.M. (2011). *Mentha arvensis* Linn. A review article. J. Med. Plants Res..

[176] Jain V., Muruganathan G., Deepak M., Vishwanatha L.G., Manohar D. (2011). Isolation and standardization of various phytochemical constituents from methanolic extracts of fruit rinds of *Punica granatum*. Nat. Med..

[177] Mehta D., Mehta M. (2012). *Punica granatum* L. (Punicaceae): Lifeline for Modern Pharmaceutical Research. J. Ethnopharmacol..

[178] Jayaprakash A. (2017). *Punica granatum*: A review of phytochemicals, antioxidant and antimicrobial properties. J. Acad. Ind. Res..

[179] Keyrouz E., El Feghali P.A.R., Jaafar M., Nawas T. (2017). *Malva neglecta*: A natural inhibitor of bacterial growth and biofilm formation. J. Med. Plants Res..

[180] Seyyednejad S.M., Koochak H., Darabpour E., Motamedi H. (2010). A survey on *Hibiscus rosa-sinensis*, *Alcea rosea* L and *Malva neglecta* Wall. as antibacterial agents. Asian Pac. J. Trop. Med..

[181] Saleem U., Akhtar R., Anwar F., Shah M.A., Chaudary Z., Ayaz M., Ahmad B. (2012). Neuroprotective potential of *Malva neglecta* is mediated via down regulation of cholinesterase and modulation of oxidative stress markers. Metab. Brain Dis..

[182] Saleem U., Khalid S., Zaib S., Anwar F., Ahmad B., Ullah I., Zeb A., Ayaz M. (2020). Phytochemical analysis and wound healing studies on ethnomedicinally important plant *Malva neglecta* Wall. J. Ethnopharmacol..

[183] Radha S.P., Pundir A. (2019). Review of Ethnomedicinal plant: *Trillium govanianum* Wall. ex D. Don. Int. J. Theor. Appl. Sci..

[184] Vishnukanta A.C., Rana A. (2008). *Melia azedarach*: A phytopharmacological review. Pharmacogn. Rev..

[185] Rani M., Suhag P., Kumar R., Singh R., Kalidhar S.B. (1999). Chemical component and biological efficacy of *Melia azedarach* stems. J. Med. Aromat. Plant Sci..

[186] Merra P.S., Kalidhar S.B. (2003). Phytochemical investigation of *Melia azedarach* leaves. J. Med. Aromat. Plant Sci..

[187] Kumari S., Anmol, Bhatt V., Suresh P.S., Sharma U. (2021). *Cissampelos pareira* L. A review of its traditional uses, phytochemistry and pharmacology. J. Ethnopharmacol..

[188] Singh S., Nishteswar K. (2013). Review on *Cissampelos pareira* and *Cyclea peltata* (Patha Dwaya)—Phyto-Pharmacological Perspectives. Int. J. Ayurvedic Med..

[189] Alqasoumi S.I., Basudan O.A., Al-Rehaily A.J., Abdel-Kader M.S. (2014). Phytochemical and pharmacological study of *Ficus palmata* growing in Saudi Arabia. Saudi Pharm. J..

[190] Maurya R., Sathiamoorthy B., Mundkinajeddu D. (2007). Flavonoids and phenol glycosides from *Boerhavia diffusa*. Nat. Prod. Res..

[191] Ferreres F., Sousa C., Justin M., Valentao P., Andrade P.B., Llorach R., Rodrigues A., Seabra R.M., Leitao A. (2005). Characterization of the phenolic profile of *Boerhaavia diffusa* L. by HPLC-PAD-MS/MS as a tool for quality control. Phytochem. Anal. Int. J. Plant Chem. Biochem. Tech..

[192] Pereira D.M., Faria J., Gaspar L., Valentao P., Andrade P.B. (2009). *Boerhaavia diffusa*: Metabolite profiling of a medicinal plant from nyctaginaceae. Food Chem. Toxicol..

[193] Kapil S.P., Sanjivani R.B. (2016). Ethnomedicinal uses, phytochemistry and pharmacological properties of the genus *Boerhavia*. J. Ethnopharmacol..

[194] Chaudhary A.K., Ahmad S., Mazumder A. (2011). *Cedrus deodara* (Roxb.) Loud. A Review on its Ethnobotany, Phytochemical and Pharmacological Profile. Pharmacogn. Res..

[195] Bahadori M.B., Sarikurkcu C., Kocak M.S., Calapoglu M., Uren M.C., Ceylan O. (2017). *Plantago lanceolata* as a source of health-beneficial phytochemicals: Phenolics profile and antioxidant capacity. Food Biosci..

[196] Adom M.B., Taher M., Mutalabisin M.F., Amri M.S., Kudos A.M.B., Wan Sulaiman M.W.A., Sengupta P., Susanti D. (2017). Chemical constituents and medical benefits of *Plantago major*. Biomed. Pharmacother..

[197] Sharma N., Pathania V., Singh B., Gupta R.C. (2012). Intraspecific variability of main phytochemical compounds in *Picrorhiza kurroa* Royle ex Benth. from North Indian higher altitude Himalayas using reversed phase high-performance liquid chromatography. J. Med. Plants Res..

[198] Vasas A., Orban-Gyapai O., Hohmann J. (2015). The Genus *Rumex*: Review of traditional uses, phytochemistry and pharmacology. J. Ethnopharmacol..

[199] Huq A.K.M.M., Jamal J.A., Stanslas J. (2014). Ethnobotanical, Phytochemical, pharmacological and toxicological aspects of *Persicaria hydropiper* (L.) Delarbre. Evid.-Based Complement. Altern. Med..

[200] Adams S.J., Kuruvilla G.R., Krishnamurthy K.V., Nagarajan M., Venkatasubramanian P. (2013). Pharmacognostic and phytochemical studies on Ayurvedic drugs Ativisha and Musta. Braz. J. Pharm..

[201] Ahmad H., Ahmad S., Shah S.A.A., Latif A., Ali M., Khan F.A., Tahir M.N., Shaheen F., Wadood A., Ahmad M. (2017). Antioxidant and anticholinesterase potential of diterpenoid alkaloids from *Aconitum heterophyllum*. Bioorgan. Med. Chem..

[202] Nisar M., Obaidullah, Ahmad M., Wadood N., Lodhi M.A., Shaheen F., Choudhary M.I. (2009). New diterpenoid alkaloids from *Aconitum heterophyllum* Wall: Selective butyrylcholinestrase inhibitors. J. Enzym. Inhib. Med. Chem..

[203] Wani Z.A., Pant S. (2021). *Aconitum heterophyllum* Wall. ex Royle: An Endemic, Highly Medicinal and Critically Endangered Plant Species of Northwestern Himalaya in Peril. Curr. Tradit. Med..

[204] Bento C., Concalves A.C., Silva B., Silva R.S. (2020). Peach (*Prunus persica*): Phytochemicals and health benefits. Food Rev. Int..

[205] Vlase L., Mocan A., Hanganu D., Benedec D., Gheldiu A. (2014). Comparative study of polyphenolic content, antioxidant and antimicrobial activity from *Galium* species (Rubiaceae). Dig. J. Nanomater. Biostruct..

[206] Morimoto M., Tanimoto K., Sakatani A., Komai K. (2002). Antifeedant activity of an anthraquinone aldehyde in *Galium aparine* L. against *Spodoptera litura* F. Phytochemistry.

[207] Al-Snafi A.E. (2018). Chemical constituents and medicinal importance of *Galium aparine*: A Review. Indo Am. J. Pharm. Sci..

[208] Tawfeek N., Mahmoud M.F., Hamdan D.I., Sobeh M., Farrag N., Wink N., El-Shazly A.M. (2021). Phytochemsirty, pharmacology and medicinal uses of plants of Genus *Salix*: An updated review. Front. Pharmacol..

[209] Chakraborthy G.S. (2009). Evaluation of immunomodulatory activity of *Aesculus indica*. Int. J. PharmTech Res..

[210] Zahoor M., Shafiq S., Ullah H., Sadiq A., Ullah F. (2018). Isolation of quercetin and mandelic acid from *Aesculus indica* fruit and their biological activities. BMC Biochem..

[211] Al-Snafi A.E. (2017). A review of *Dodonaea viscosa*: A potential medicinal plant. IQSR J. Pharm..

[212] Ahmad M., Butt M.A., Zhang G., Sultana S., Tariq A., Zafar M. (2018). *Bergenia ciliata*: A comprehensive review of its traditional uses, phytochemistry, pharmacology and safety. Biomed. Pharmacother..

[213] Riaz M., Zia-ul-Haq M., Jaffar H.Z.E. (2013). Common mullein, pharmacological and chemical aspects. Braz. J. Pharmacogn..

[214] Sayyed A., Shah M. (2014). Phytochemistry, pharmacological and traditional uses of *Datura stramonium* L. review. J. Pharmacogn. Phytochem..

[215] Asgarpanah J., Mohajerani R. (2012). Phytochemistry and pharmacologic properties of *Urtica dioica* L. J. Med. Plants Res..

[216] Chashoo I.A., Kumar D., Bhat Z.A., Khan N.A., Kumar V., Nowshehri J.A. (2012). Antimicrobial activities of *Sambucus wightiana* Wall. ex Wight & Arn. J. Pharm. Res..

[217] Wang X., Shi H.M., Li X.B. (2010). Chemical Constituents of Plants from the Genus *Viburnum*. Chem. Biodivers..

[218] Muhammad A., Ghaisuddin, Anwar S., Naveed M., Ashfaq A.K., Bina S.S. (2012). Evaluation of *Viburnum grandiflorum* for its in-vitro pharmacological screening. Afr. J. Pharm. Pharmacol..

[219] Borlinghaus J., Albrecht F., Gruhlke M.C.H., Nwachukwu I.D., Slusarenko A. (2014). Allicin: Chem & Biol Prop. Molecules.

[220] Debnath B., Singh W.S., Das M., Goswami S., Singh M.K., Maiti D., Manna K. (2018). Role of plant alkaloids on human health: A review of biological activities. Mat. Today Chem..

[221] Habtemariam S. (2016). Rutin as a Natural Therapy for Alzheimer’s Disease: Insights into its Mechanisms of Action. Curr. Med. Chem..

[222] Ghorbani A. (2017). Mechanisms of antidiabetic effects of flavonoid rutin. Biomed. Pharmacother..

[223] Wang W., Sun C., Mao L., Ma P., Liu F., Yang J., Gao Y. (2016). The biological activities, chemical stability, metabolism and delivery systems of quercetin: A review. Trends Food Sci. Technol..

[224] Baghel S.S., Shrivastava N., Baghel R.S., Agarwal P., Rajput S. (2012). A review of Quercetin: Antioxidant and anticancer properties. World J. Pharm. Pharm. Sci..

[225] Wal P., Wal A., Sharma G., Rai A.K. (2011). Biological activities of Lupeol. Syst. Rev. Pharm..

[226] Jhonston G.A.R., Chebib M., Duke R.K., Fernandez S.P., Hanrahan J.R., Hinton T., Mewett K.N. (2009). Herbal Products and GABA Receptors. Encyclopedia of Neuroscience.

[227] Shukla R., Panday V., Vadnera G.P., Lodhi S. (2019). Role of Flavonoids in Management of Inflammatory Disorders. Bioactive Food as Dietary Interventions for Arthritis and Related Inflammatory Diseases.

[228] Wang S.C., Lu M.C., Chen H.L., Tseng H., Ke Y., Wu Y.C., Yang P. (2009). Cytotoxicity of calotropin is through caspase activation and downregulation of anti-apoptotic proteins in K562 cells. Cell Biol. Int..

[229] Cheng L., Xia T., Wang Y., Zhou W., Liang X., Xue J., Shi L., Wang Y., Ding Q., Wang M. (2014). The anticancer effect and mechanism of α-hederin on breast cancer cells. Int. J. Oncol..

[230] Guo Y., Liu Y., Zhang Z., Chen Z., Zhang Z., Tian C., Liu M., Jiang G. (2020). The antibacterial activity and mechanism of action of Luteolin against *Trueperella pyogenes*. Infect. Drug Resist..

[231] Qi S., Quan L.Q., Cui X.Y., Li X., Zhao X.D., Li R.T. (2019). A natural compound obtained from *Valeriana jatamansi* selectively inhibits glioma stem cells. Oncol. Lett..

[232] Imran M., Salehi B., Sharifi-Rad J., Aslam T.G., Saeed F., Imran A., Shahbaz M., Fokou P.V.T., Arshad M.U., Khan H. (2019). Kaempferol: A Key Emphasis to its Anticancer Potential. Molecules.

[233] Zhang J., Liu N., Zhang J., Liu S., Liu Y., Zheng D. (2005). PKC*δ* protects human breast tumor MCF-7 cells against tumor necrosis factor-related apoptosis-inducing ligand-mediated apoptosis. J. Cell. Biochem..

[234] Borris R.P. (1996). Natural products research: Perspectives from a major pharmaceutical company. J. Ethnopharmacol..

[235] Pieters L., Vlietinck A.J. (2005). Bioguided isolation of pharmacologically active plant components, still a valuable strategy for the finding of new lead compounds?. J. Ethnopharmacol..

[236] Said A., Naeem N., Siraj S., Khan T., Javed A., Rasheed H.M., Sajjad W., Shah K., Wahid F. (2020). Mechanisms underlying the wound healing and tissue regeneration properties of Chenopodium album. 3 Biotech.

[237] Birdane F.M., Cemek M., Birdane Y.O., Gülçin I., Büyükokuroğlu E. (2007). Beneficial effects of vulgare ethanol-induced acute gastric mucosal injury in rat. World J. Gastroenterol..

[238] McGaw L.J., Eloff J.N., Katerere D.R., Luseba D. (2010). Methods for evaluating efficacy of ethnoveterinary medicinal plants. Ethnoveterinary Botanical Medicine: Herbal Medicines for Animal Health.

